# Health Innovation in Patty Products. The Role of Food Neophobia in Consumers’ Non-Hypothetical Willingness to Pay, Purchase Intention and Hedonic Evaluation

**DOI:** 10.3390/nu11020444

**Published:** 2019-02-20

**Authors:** Zein Kallas, Mauro Vitale, José Maria Gil

**Affiliations:** 1 Centre for Agro-food Economy & Development (CREDA-UPC-IRTA), 08860 Castelldefels, Spain; chema.gil@upc.edu; 2 Institute of Agrifood Research and Technology (IRTA), Product Quality Program, 17121 Monells, Spain; mvitale@metalquimia.com

**Keywords:** food innovations, hedonic evaluation, non-hypothetical discrete choice experiment, food neophobia, patty, untapped pig

## Abstract

Consumers’ personality traits are key factors in understanding consumers’ choice and acceptance for health innovations in food products, in particular, food neophobia (FN). The patty product as a traditional pork product (TPP) with two innovative traditional pork products (ITPP) from the untapped pig breed (Porc Negre Mallorquí) in Spain were analysed. Patties were enriched with Porcini (*Boletus edulis*) using the claim “enriched with a natural source of dietary fiber Beta glucans that may contribute to improve our defence system” (ITPP1) and enriched with blueberries (*Vaccinium corymbosum*) using the claim “enriched with a natural source of antioxidant that may help to prevent cardiovascular diseases” (ITPP2). Two non-hypothetical discrete choice experiments were applied to investigate the importance of FN in consumers’ purchase intention (PI) and willingness to pay (WTP) before and after tasting the products. Results showed that the TPP and the ITPP2 received higher than expected PI and WTP. However, after tasting the products, consumers exhibited lower WTP for all ITPP showing the prevalence of the sensory experience on health innovation. The FN was highly related to WTP before the hedonic evaluation. However, it turned out to be non-significant, showing a homogenising role of the sensory experience in reducing the FN impact.

## 1. Introduction

Food health innovations are becoming determinant factors affecting consumers’ food choice. Consumers’ preference and acceptance of food innovations are multidimensional and rely on a mixture of the product intrinsic and extrinsic cues, expectations, socio-economic characteristics, and attitudes [1,2]. Personality traits [3], in particular, food neophobia (FN) [4,5] is one of the most relevant key factors. FN is an individual-specific trait [6] that describes human unwillingness to consume unfamiliar food [7]. Although FN has been examined extensively in recent decades [8], the study of the relationship between FN and consumers’ food purchase intention and willingness to pay in a “real” context still limited [9]. In general term, research that associated the FN trait to food choice have used hypothetical survey frameworks. Accordingly, consumers are asked about their purchase intention (PI) and preferred food choice in which the product price did not influence their answer. Surveys may, in general, suffer from the hypothetical bias which reflects the old saying: “there is a difference between saying and doing” [10]. Allowing surveys to be consequential to respondents using non-hypothetical frameworks is an ex ante approach that may reduce this bias [10]. 

On the basis of the Ajzen’s theory of planned behaviour, ref. [11] analysed in a hypothetical survey the PI of organic food and FN in Taiwan. This study considered the price in an aggregated approach by estimating an interaction term between price and FN. Results showed non-significant associations. Ref. [12] analysed the PI with a hedonic evaluation test for health claims using FN as moderator. Results showed a secondary role of FN in constructing the consumers’ final purchase decision. Ref. [13] highlighted the importance of price in the consumer purchase decision and stated that consumers may purchase the product they like less due to its lower price. Accordingly, when consumers face a purchase situation they may choose on the basis of some product cues or information (health, convenience and price …) but “actually prefer the food not chosen”. Therefore, consumers may trade off and compromise some products’ attributes if the product is cheaper.

Within this approach, it is relevant to analyse the role of FN when associated to the PI and willingness to pay (WTP) when the price information is available, in particular in non-hypothetical framework. Ref. [14] analysed the importance of FN in a more realistic PI for local innovative food products, introducing the product price as a relevant attribute but using a hypothetical experimental approach. Ref. [15] in a non-hypothetical experimental auction have associated the FN trait to consumers’ willingness to pay for traditional salami under a different information context and tasting with non-significant results. The hedonic evaluation is a relevant factor tightly related to FN traits. Consumers with high FN tend to exhibit low expected liking for unfamiliar products which is associated to the expected unpleasant taste [16]. Ref. [17] in a non-hypothetical multiple price list approach have found that high levels of neophobia negatively affect the WTP for insect-based products. In all cases, there is lack in consumers’ studies that relate FN to the consumers’ non-hypothetical purchase intention and willingness to pay using the choice experiment approach that simulate real purchasing situations with “real” money and products.

In this context, the objective is to analyse the impact of the FN trait on consumers’ non-hypothetical PI and WTP using the non-hypothetical discrete choice experiment (NH-DCE) method simulating real purchasing scenarios before and after tasting the product. For that purpose, we created several non-hypothetical purchase scenarios using a D-optimal choice design by involving real products and money to ensure the consequentiality of the consumers’ decision. The patty product as a traditional pork product (TPP) with two innovative traditional pork products (ITPP) including health innovations and claims were used. The products were obtained from the untapped and local pig breed in Spain (Porc Negre Mallorquí) that fit within the measures that aim to protect the local, autochthonous and untapped pig breeds by creating added-value products that meet consumers’ preferences and market demand [18]. The first innovation was to enrich the patties with Porcini (*Boletus edulis*) as a natural source of dietary fibre (Beta glucans) using the claim “enriched with a natural source of dietary fibber that may contribute to improve our natural defence system”. The second was to enrich the patties with blueberries (*Vaccinium corymbosum*) as a natural source of antioxidants using the claim “enriched with a natural source of antioxidant that may help to prevent cardiovascular diseases” [19,20,21].

This paper contributes to the existing literature of consumers’ FN in two aspects. Firstly, at the methodological level, it contributes to the very scarce literature that relates the FN to the willingness to pay and purchase intention, using real shopping scenarios with tasting experience. Before purchasing a new product, the extrinsic attributes are the key factors that infer its quality [22]. However, when tasted, consumers are able to construct an experience of quality that may affect their purchasing decision. To our knowledge this is the first study applying the NH-DCE with labelled choice design that associate the FN to consumers’ preferences and acceptance. Secondly, at an empirical level, as commented on by [23], it contributes to re-testing the reliability of the FN scale and critical assessment of items still valuable. While the FN scale was tested and validated in several languages, this study is an opportunity to make the scale accessible in the Catalan language.

## 2. Material and Methods

Our theoretical approach relies on the expectancy-disconfirmation model [24] and in part on the total food quality model [25]. It involves a comparison between the cognitive state (expected PI and WTP) prior to an event (hedonic evaluation test) and the subsequent cognitive state (experienced PI and WTP) after the event. According to this approach, many characteristics of a food product cannot be discovered before purchase. Consumers develop expectations about its quality when making a food choice [22] and they rely on its extrinsic attributes to deduce its quality. Once the product is consumed, these expectations may change. If the experience matches the expectation, confirmation occurs, which results in satisfaction. If there is a mismatch, a positive disconfirmation may occur if experience improves expectations and a negative disconfirmation may occur if experience worsens expectations. The experiment was carried out in three-steps:

1. Firstly, in an initial questionnaire, a NH-DCE is applied to assess consumers’ non-hypothetical “expected” PI and WTP.
2. Secondly, in a further questionnaire, an informed hedonic evaluation test of the same products from the first step was carried out.
3. Thirdly, the initial questionnaire with the same NH-DCE was repeated to assess consumers’ non-hypothetical “experienced” PI and WTP and to analyse the role of the hedonic evaluation in determining the consumers’ final decision towards the proposed innovations and its impact on the FN trait.

### 2.1. Consumers’ Sample

Data was collected from 121 consumers having purchased and consumed the patty product during the last month and stratified in terms of gender and age according to the population of Catalonia. A quota sampling procedure was used. The experiment was conducted in Barcelona (Spain) during February 2017. The Catalan region was selected due to the closeness of this region to Mallorca (the origin of the untapped pig breed) as it was considered the closest biggest market to the proposed added-value patties. The sample size was adjusted to what is commonly used in the literature of consumers’ acceptance studies [26]. The budget and time constraints, the availability of real products in the proposed purchasing scenario, and the processing time were highly limiting factors to increase the sample size. Therefore, results should be taken with care if extrapolated to population by highlighting the exploratory nature of this study.

Consumers were motivated and economically compensated to participate with 25€ per respondent. We clearly explain to them at the beginning that the money they would get at the end of the experiment is the monetary equivalent to the time they spend by participating in the experiment and, therefore, they should consider it as part of their available income and not as a gift [27].

Each experimental session lasted approximately 1.5 h. Table 1 represents a summary of the sample description. The experiment was approved by the ethic committee of the Centre for Agro-food Economy and Development and was conducted according to the ethical principles expressed in the Declaration of Helsinki with a specific care on protecting personal information according to the new European regulations. Before conducting the experiment, the participants signed a consent form and received an explanation of the experiment which was read to them aloud and projected using PowerPoint before starting in each case study.

### 2.2. Products and Innovations

The first ITPP1 in Spain was obtained by enriching the patties with Porcini (*Boletus edulis*) as a natural source of dietary fibre (Beta glucans). The second one (ITTP2) was obtained by enriching the patty with blueberries (*Vaccinium corymbosum*) as a natural source of antioxidants. The main criteria used in the election of each innovation were: (a) the relevance of the innovation in tackling with the most relevant consumers’ health concerns. The proposed innovations may contribute to disease prevention related to preventing cardiovascular diseases related to the consumption of natural antioxidant [28], (b) the capacity to include the innovations and produce the ITPP at small scale for the experimental performance, (c) the ability to afford the production cost due to budget constraints and (d) the availability of meat or products taking into account the limited resources of the untapped breeds.

The TPPs and the ITPPs produced from the untapped breed were compared with two additional products obtained from commercial pig breeds. The first product was with “conventional quality” (CONV) that met the standards and the minimum requirements of the production process with relatively “normal” or low prices. The second product was with “premium quality” (PREM) that goes beyond the minimum standard and quality requirement with relatively higher prices. Both the CONV and the PREM products were produced to ensure homogeneity in the production qualities when compared to the TPP and the ITPPs.

### 2.3. Analysing the Non-Hypothetical Purchase Intention (PI) and Willingness To Pay (WTP) for Innovative Pork Products

We used the Non-Hypothetical Discrete Choice Experiment (NH-DCE) methodology to analyse consumers’ PI and WTP, measured before and after a hedonic evaluation test. The previously defined pork products (TPP, ITPP CONV, and PREM) were jointly presented to respondents in an array of repeated simulated purchase situations (cards) at different price levels. The “NONE” option (i.e., neither of them) was also included to be consistent with the demand theory and to make the choice task more realistic as this option is available when shopping. Respondents were asked to select the product that they would purchase for sure in a simulated market situation, thereby revealing their preference for certain characteristics of the products. Each product type was assigned four price levels. The product format was a tray of 250 g with 2 patties. The price levels for the TPP were (3.00 €, 3.75 €, 4.50 € and 5.25 €), for the ITPP1 (3.00 €, 3.75 €, 4.50 € and 5.25 €), for the ITTP2 (3.00 €, 3.75 €, 4.50 € and 5.25 €), for the CONV (2.00 €, 2.50 €, 3.00 € and 3.50 €) and for the PREM (3.00 €, 3.75 €, 4.50 € and 5.25 €). For the CONV and PREM products, the price levels were identified by analysing the lowest and highest values in the market place. For the TPP, ITPP1 and ITPP2, the price vectors were not determined by the actual prices of the products because they are not yet commercialized. However, they were identified by unobserved demand curves on the basis of prior knowledge concerning the additional production cost and the expected maximum willingness to pay for these types of product [29].

We defined eight purchase situations by means of a D-optimal fractional factorial labelled choice design [30] with D optimality of 83.34% using the Ngene software [31]. The choice sets and the questionnaire are available in the attached Supplementary Data File. To ensure the non-hypothetical nature of the experiment, before the NH-DCE questions, participants were informed that one product will be delivered from a randomly selected purchase situation. Consumers were also informed that all the products are “real” and produced to be “sold” at the end of the experiment. The non-hypothetical nature of the experiment implies an interchange of money and preferred products for all participants if they agreed to participate. Firstly, we randomly select which NH-DCE is binding (i.e., before or after the hedonic evaluation) by having one of the participants draw a number out of an envelope from 1 to 2. Secondly, we randomly select which choice situation is binding by having one of the participants draw a number out of an envelope from 1 to 8. Once the randomly purchase situation is identified, consumers were requested to look for their answers. If the NONE option was selected, no product is delivered and consumers were invited to leave the experiment room. If consumers selected any other product, they were asked to pay its posted price and to take their selected product.

The DCE relies on Lancaster’s theory of value [32] and on the random utility theory (RUT) of [33]. The individuals choose among the product, in a purchase situation, according to a utility function with two main components: a systematic (observable) component and a random error term (non-observable):

(1) $$U_{jnt}=V_{jnt}+\varepsilon_{jnt}$$

where *U_jnt_* is the utility of product j to subject n in choice set t, $V_{jnt}$ is the systematic component of the utility and $\varepsilon_{jnt}$ is a stochastic term. In our case, the utility function for product j can be expressed as:

(2) $$V_{jnt}=\beta_{j}.ASC_{jnt}+\sum_{j=1}^{J}\alpha_{j}.P_{jnt}$$

where j are the TPP, ITPP1, ITPP2, CONV, and PREM products. $P_{jnt}$ is the *j*th product’s price for consumer *n*, $\beta_{j}$ are the coefficients of the alternative specific constant for each product *j* and consumer which represents the marginal utility of the product *j*. $\alpha_{j}$ are the coefficients representing the effect of the *j*th product price on the utility for another *j*th product.

To predict the subjects’ choice for a product, we used the random parameters logit (RPL) model. In this case, the coefficient vector of the ASC (Alternative Specific Constant) is decomposed as $\beta_{j}={\bar{\beta }}_{j}+\sigma_{j}\;\lambda_{nt}$, where ${\bar{\beta }}_{j}$ is the estimated mean of the ASC and $\sigma_{j}$ is the standard deviation of the marginal distribution of ${\bar{\beta }}_{j}$ and $\lambda_{nt}$ is a random term assumed to be normally distributed with mean zero and unit standard deviation. The price coefficients are considered as fixed parameters to ensure that the estimated total WTP is normally distributed. The WTP of a product j versus the baseline alternative NONE is calculated as the negative ratio of the ASC coefficient to the price coefficient of the same product *j* [30]:

(3) $$WTP_{\mathrm{Product}\;j\;\mathrm{Vs}.\;\text{No-option}}=-\left(\frac{\frac{d}{d\;ACS_{jnt}}\beta_{j}.{ASC}_{\;jnt}}{\frac{d}{d\;P_{jnt}}\alpha_{j}.P_{jnt}}\right)=-\left(\frac{\beta_{j}}{\alpha_{j}}\right)\;=\left(\frac{\beta_{\mathrm{Product}\;j}}{\alpha_{\mathrm{price}\;j}}\right)$$

Finally, the Krinsky and Robb parametric bootstrapping method was applied to calculate the confidence intervals of the WTPs with 1000 random repetitions using NLOGIT 5.0 software.

### 2.4. Measuring the Food Neophobia (FN)

Recently, ref. [23] carried out a review in which they reported 13 instruments to measure FN. While they did not identify a superior measurement tool, they mentioned that the Food Neophobia Scale (FNS) developed by [4] is currently the most widely used psychometric tool to investigate FN and to predict consumers’ response towards new food products [5,34]. This scale consists of five positive and five negative statements (Table 2) towards different situations of food consumption, using a 9-point Likert scale with the following categories: “disagree very strongly”, “disagree strongly”, “disagree moderately”, “disagree slightly”, “neutral: do not agree nor disagree”, “agree slightly”, “agree moderately”, “agree strongly” and “agree very strongly”. The original scale version was translated into the Catalan language and tested for the comprehension of the items in a pilot sample of about 10–16 consumers. Some items and words have been adapted and improved to ensure comprehension. The internal consistency and validity of the scale was measured by Cronbach’s Alpha and principal component analysis (PCA). The individual FN score was calculated by summing all the ratings of positive statements with reversed scores of negative statements [35].

To assess the FN association with the non-hypothetical PI and WTP, we carried out two-step cluster analysis (TSCA) rather than splitting the FN scores into tertiles as proposed by [35]. The classification procedure was based on the log-likelihood measure that places a probability distribution on the FN score to identify the optimum number of the cluster by identifying the corner change in the values and thus the “natural” grouping of consumers. The silhouette coefficient was used to test the cluster’s quality extraction.

### 2.5. Measuring Consumers’ Expected Liking and the Informed Tasting Test

The expected liking of the TPP, ITPP, CONV and PREM was obtained using the direct numerical probability elicitation method [36]. Consumers were asked to state their expected liking in a probabilistic way ranging from “0%” where there is no chance that they would like the product to 100% where they are sure that they would like the product. Later an informed hedonic evaluation test of the TPP, ITPP, CONV and PREM was carried out following the protocol presented in [37]. Samples were grilled at 165 °C to an internal temperature of 70–75 °C and cut into quarters and kept at 25 °C until tasting. The overall acceptability of the products j (TPP, ITPP, CONV and PREM) was assessed using the 9-point hedonic scale ranging from “I extremely dislike” to “I extremely like”. The products’ valuation was conducted in individual booths. Consumers were instructed to eat unsalted toasted bread and drank mineral water between samples [38]. Each product sample was assigned three digit random numbers and presented to consumers in random order according to a randomized complete-block design. They were asked to taste the products to taste with an information sheet that should be carefully read. The information that describe each product was similar to the posted on the products in the purchasing scenarios regarding the innovation type, the health claim and the pig breed. They were asked to taste the products and identify which product they are tasting (informed liking).

## 3. Results

### 3.1. The Non-Hypothetical WTP and PI for Food Innovations in Pork Products

Two RPL models were estimated before and after the hedonic evaluation test (Table 3). Results showed that at a 99% confidence level, we can reject the null hypothesis that all coefficients are jointly equal to zero with a highly acceptable goodness of fit (McFadden’s pseudo-R^2^). The positive/negative sign of the coefficients implies higher/lower levels of utility associated with the products, and thereby with their characteristics. The model estimates showed that almost all coefficients are statistically significant. All the estimated standard deviations of the random coefficients (ASCs) were highly significant, confirming the suitability of the model. However, estimates before and after tasting the products cannot be compared due to the scale parameter. Comparisons can only be evaluated at the WTP level using the Poe test [39].

Following Equation (3), we estimated the expected and experienced WTPs. Results (Table 4) showed a positive expected preference, in general, of the new products proposed from the untapped pig breeds and the innovations. The expected WTP showed the highest values for the TPP compared to CONV. However, the expected WTP for the ITPP1 (patties enriched with natural source of dietary fibber) was similar to the CONV product and the expected WTP for the TPP and the ITPP2 (patties enriched with natural antioxidant) was similar to the PREM product. 

After the hedonic evaluation, the expected WTP (3.48 €) for the TPP were confirmed by the experienced WTP (3.60 €) where a non-significant difference was identified. The hedonic evaluation, in this case, had no significant impact on consumers’ WTP and preference change. For the ITPP, results showed that after the hedonic evaluation the expected WTP was negatively disconfirmed (decreased significantly). Consumers expected more from the proposed innovations in terms of taste and therefore the hedonic evaluation played a relevant role in determining the final preference patterns. In this same context, consumers expected more for the PREM and preference was only confirmed in the case of the CONV product.

The PI in Table 4 was also calculated by summing, for each product, how many times the product were chosen as the most preferred one in all purchase scenarios. Analysing the expected PI, results showed a relatively low rate of preference for the TPPs and the ITPPs compared to the CONV, PREM and NONE alternatives. However, the estimated share of all products from the untapped breeds showed a potential preference at market level. The expected PI for the TPP jointly with the ITPPs had 44.10% of the total selections.

The impact of the hedonic evaluation on the expected PI was heterogeneous. The expected PI for the TPP increased (positive disconfirmation). However, the purchase intention remained unchanged for the innovative products (ITPP1 and ITPP2). Comparing the impact of the hedonic evaluation on the WTP and the PI, results (Table 4) showed some foreseen divergence. This is because the fact that the product with the highest PI does not necessary imply the highest WTP [30], since the products were presented with different price levels. This outcome shows the importance of considering the product’s own price and that of their competing counterparts when analysing preferences and understanding consumers’ reaction to food innovations.

### 3.2. The Food Neophobia Trait

The internal consistency of the scale (Cronbach‘s Alpha) was 0.847, demonstrating highly acceptable validity level. Before estimating the FN score of each respondent, we first checked for the factor structure of the FNS using PCA. Two factors were identified. The low food neophobic factor (F1) and the high food neophobic factor (F2). In general, the PCA correctly separated the positive items from the negative ones, thereby, confirming the suitability of the FNS to describe the FN trait. However, not all items were classified as expected within the PCA. Item 9 and the item 8 were not well associated to the expected factors. Therefore, a new PCA was estimated after discarding both items whose results are presented in Table 5. The goodness of fit and the consistency of this PCA significantly increased after dropping items 8 and 9.

As previously commented, for the estimation of consumers FN, the individual FN scores were calculated by summing all the ratings of positive statements with reversed scores of negative statements. Results (Table 6) showed relatively low FN levels. Results of the two-step cluster analysis showed three natural clustering structures (Table 6): the low neophobic cluster (Low FN C1), the neutral neophobic cluster (Average FN C2) and the high neophobic cluster (High FN C3). The average silhouette measure of cohesion and separation showed a good cluster quality with 0.7 value.

#### 3.2.1. FN Association with the Non-Hypothetical WTP and PI

The association of consumers’ FN and the non-hypothetical WTP and PI are presented in Table 7. Comparisons were reported only between low and high FN clusters to better highlight the FN role. Focusing on the TPP, low FN consumers showed higher non-hypothetical PI compared to the high FN ones. However, non-significant differences were found. Analysing the expected PI of the innovations (ITPPs), a clear tendency was identified where low FN consumers exhibited higher percentages than the high FN ones with statistically significant differences. Food neophobic consumers showed some reluctance regarding the innovative pork introduced in the patty product. 

Results showed that the low FN consumers showed the highest expected and experienced PI for ITPP and the TPP compared to the high FN consumers. However, significant differences were found for the expected and experienced PI of the ITPP2 (31.77% compared to 14.32% and 27.60% compared to 16.76%, respectively) but only for the expected PI of the ITPP1 (12.50% compared to 7.27%). An additional finding is related to changes that occurred to the expected PI after the hedonic evaluation test and their relation to the FN. The hedonic evaluation mitigated the difference of the experienced PI between the low FN and the high FN consumers for the ITPP2.

Results also showed that, in the proposed purchase situations, consumers with high FN tended to select more the option “neither of them” than the low FN ones. After the hedonic evaluation this trend was maintained. However, the difference in the percentage of times that the NONE option was selected turned to be relatively lower (i.e., difference between 3.65% and 17.50% compared to the difference between 4.10% and 16.76%).

The FN trait was also related to the non-hypothetical WTP (Table 7). Results showed that the relation between the FN and WTP was highly significant before the hedonic evaluation, while it turned out to be non-significant after the hedonic evaluation test, showing a homogenising role of the tasting in reducing the FN impact. In all cases, compared to the IP results, the WTP association with the FN was able to extract more significant relations, in particular at the expectation level. This result showed again the relevance of the price attribute when introduced in the analysis of the relation of FN with food choice.

#### 3.2.2. The Impact of FN on the Probabilistic Expected and Informed Liking

The FN was associated with the direct numerical probability expected liking. Focusing on the traditional products and innovations from the untapped breed (TPP, ITPP1 and ITPP2), results (Table 8) showed a clear pattern. Consumers with low FN exhibited higher expected liking probabilities for the TPP and ITPP than the high FN with statistically significant difference. Furthermore, non-significant differences were found for the CONV and PREM products.

This trend was maintained for the informed liking scores were low FN consumers exhibited higher acceptance of the products from the untapped breed. However, non-significant results were found for the the ITPP1 (patties with Porcini (*Boletus edulis*) as a natural source of dietary fibre). In all cases, the informed liking scores were lower for the proposed innovation than the other products showing a reluctance despite their “theoretical” health benefit. Only the traditional pork product (TPP) received the highest acceptance level showing a clear preference towards a more natural product with no added healthy compounds or ingredients when promoting the patty products.

## 4. Discussion

### 4.1. Reliability of the Adapted FN Scale

After dropping items 8 and 9, the PCA was highly significant and the percentage of the total variance explained was also acceptable compared to other FN studies [35,40]. The variance by the first and second dimension was also within the acceptable range of the FNS studies [34,35,40]. The adapted FN scale has been shown to be a valid and reliable tool to extract consumers’ FN in our analysed sample.

Discarding items from the original FNS is not new because of the potentially dissimilar interpretations of what consumers from different culture may understand from the original statements [34]. In their study, they dropped items 5 and 9 due to a misinterpretation of statements (translation or other latent problems) in a Swedish case study. They also mentioned that the FNS is not unidimensional since the understanding of some items does not express the same phenomenon depending on the sample analysed. They even realized that a model based on 6 items only (1, 3, 4, 6, 7, and 10) may be applicable to understand consumer FN and reaction to novel food products when compared between countries (US, Sweden and Finland) as was applied in [41] who included only items that were deemed culturally suitable for Kenyan consumers. Ref. [11] used only five items of the FNS (1, 2, 3, 7, 9) following [42]. Ref. [43] also used a reduced FNS form using 5 items obtaining good reliability of the scale. In any case, ref. [23] in their review stated that excluding items from the FNS may improve the FN structure when used in several samples and recommended a prior evaluation of the content of items in the FNS and using only those that are relevant in each context.

### 4.2. The Importance of FN in the Analysis of Consumers’ Preference and Acceptance

The FN was significantly associated to the expected PI when innovations consisted of enriching the products with unfamiliar components such as those introduced in our case study with their respective health claim. There was a significant tendency regarding the low FN consumers in revealing higher PI toward innovations due to the relatively novel innovations in patty products. Introducing only the breed type as a differentiating attribute did not create major changes regarding the familiarity perception, since non-significant results were found for the TPP. The association of the FN with the expected WTP confirmed and accentuated the previous results. Including the price attribute in the analysis of the association of FN with consumers’ food choice with unfamiliar innovations in a non-hypothetical framework led to higher significant relation [41,44,45].

It is also important to verify that some changes occurred regarding this association after the hedonic evaluation test. Several studies showed that the significance of the FN associations can vary according to experimental environment. The tasting experience with novel and unfamiliar flavours can produce changes in preference [46,47]. Refs. [48,49] showed that tasting education and the eating experience may reduce FN, as appeared in our case study. After the hedonic evaluation, the association of the FN with the experienced PI and the experienced WTP for the ITPP1 (enriched with dietary fibber) turned out to be non-significant. The same also occurred for the experienced WTP for the ITPP2 (enriched with natural antioxidant), highlighting in this case the relevance of considering also the WTP when consumers’ purchase choice is related to FN.

Our results showed that the findings from the estimated WTP may diverge from those obtained from the PI due the relevance of the price attribute in defining consumers’ purchasing decision. The inclusion of a non-forced choice gave the consumers the opportunity to opt out in their choice [50]. Thus, the WTP is able to capture higher preference variability between food neophobic and neophilic consumers. Considering the price information in drawing consumer’s preferences is a key factor for understating the consumers’ final decision towards food innovations. Focusing on the hedonic evaluation, consumers with THE lowest FN showed the highest probabilistic expected liking for the TPP and the ITPP in comparison to consumers with the highest FN as also highlighted in other several studies [14,16,35].

## 5. Conclusions

The results showed a high non-hypothetical expected WTP and expected PI for products obtained from the unfamiliar pig breed, revealing high potential for their market penetration. However, after the hedonic evaluation, the expected WTP for the proposed innovations were negatively disconfirmed. Including the tasting experience in research that focuses on consumers WTP towards food innovation would help to understand consumers’ final food choice decision. The difference between the expected and experienced WTP towards the innovations showed high variability compared to the difference between the expected and experienced PI. Our results highlight the relevance of the additional information gathered when the price attribute is considered in a non-hypothetical framework to define consumers’ preference for food innovations.

Focusing on the proposed health innovations in patties, the FN was significantly more associated with the expected WTP than the expected PI variable. Consumers with a low FN trait exhibited higher expected WTP, in particular for the unfamiliar innovations in patty products (enriched with a natural source of dietary fiber and enriched with a natural antioxidant). However, after the hedonic evaluation, the FN association with the experienced WTP turned out to be non-significant for the innovations. This results highlight the homogenizing role of the eating experience. Furthermore, consumers with high FN tended to select more the option “neither of the products” showing a reluctance for purchasing non familiar products.

A trend appeared for the consumers with low FN who exhibited higher expected liking probabilities towards the products from the untapped pig breeds and innovations. Our study showed that the FN trait is likely to play a relevant role in defining the consumers’ liking expectations, the non-hypothetical PI, and WTP for the proposed food innovations. In any case, it would be worth considering the role of the food innovations regarding their familiarity and novelty, and a difference may appear if they consist of a reduction or an enrichment and if they are introduced in fresh or processed products when associated to FN. Future research with large samples is required to better identify more significant results regarding the introduction of untapped pig breed into the markets.

Our research showed that a market niche exists where no “add-ons” are required to improve consumers’ preferences. The TPPs and the ITPP were equally perceived as healthy products showing that the suggested innovations were not significant. Marketing strategies that promote products from the untapped pig breed should focus on the “natural” version of the product. This may allow consumers to consider the product with a special focus on the untapped pig production system.

## Figures and Tables

**Table 1:** Summary of the socio-economic and demographic variables.

Gender	Female	48.76%
	Male	51.24%
Age categories	18–29 years	12.40%
	30–39 years	21.49%
	40–49 years	26.45%
	50–59 years	22.31%
	>60 years	17.36%
Family members	Average	2.92
% with children <12 years	Yes	19.83
Number of children <12 years	Average	1.46
Household monthly net income compared to the average	Far below average	18.18%
	Below average	26.45%
	Average	32.23%
	Above average	18.18%
	Far above average	2.48%
	I don’t know	2.48%
Household monthly food expenditure compared to the average	Far below average	5.00%
	Below average	21.67%
	On average	26.67%
	Above average	38.33%
	Far above average	5.83%
	I don’t know	2.50%

**Table 2:** The Food Neophobia Scale (FNS) scale.

1. (R) I am constantly sampling new and different foods
2. I do not trust new foods
3. If I do not know what a food is, I will not try it
4. (R) I like foods from different cultures
5. Ethnic food looks weird to eat
6. (R) At dinner parties, I will try new foods
7. I am afraid to eat things I have never had before
8. I am very particular about the foods I eat
9. (R) I will eat almost anything
10. (R) I like to try ethnic restaurants

R: Reversed.

**Table 3:** Random parameters logit (RPL) models before and after the hedonic evaluation test.

β_s_	RPL	
	Expected	Experienced
	Random β_s_	
ASC-TPPβ_1_	4.77 ***	6.40 ***
ASC-ITPP1β_2_	4.00 ***	3.25 ***
ASC-ITPP2β_3_	4.64 ***	2.06 ***
ASC-CONVβ_4_	3.06 ***	2.63 ***
ASC-PREMβ_5_	4.95 ***	3.29 ***
	Non-random α_s_	
PRICE-TPPα_1_	−1.36 ***	−1.77 ***
PRICE-ITPP1α_2_	−1.27 ***	−1.25 ***
PRICE-ITPP2α_3_	−1.28 ***	−1.19 ***
PRICE-CONVα_4_	−1.12 ***	−1.01 ***
PRICE-PREMα_5_	−1.38 ***	−1.22 ***
	S.D. σ_s_ of random β_s_	
S.D. TPPσ_1_	3.31 ***	5.13 ***
S.D. ITPP1σ_2_	2.43 ***	3.48 ***
S.D. ITPP2σ_3_	2.87 ***	5.68 ***
S.D. CONVσ_4_	2.74 ***	3.95 ***
S.D. PREMσ_5_	3.52 ***	5.19 ***
Pseudo R^2^	0.33	0.45

*** *p* < 0.01.

**Table 4:** Willingness to pay (WTP) and purchase intention (PI) before and after the hedonic evaluation test.

Products	Expected Before the Informed Tasting	Experienced After the Informed Tasting
TPP (Purchase Intention, %)	14.6% ^y^	21.8% ^x^
Analysis of variance (ANOVA)	Positive Disconfirmation	
TPP (Willingness to Pay)	3.48 € ***^a^	3.60 € ***^a^
Poe test	Confirmation	
ITPP1 (Purchase Intention, %)	10.8% ^x^	10.5% ^x^
ANOVA	Confirmation	
ITPP1 (Willingness to Pay)	3.13 € ***^b^	2.59 € ***^b^
Poe test	Negative Disconfirmation	
ITPP2 (Purchase Intention, %)	18.7% ^x^	18.6% ^x^
ANOVA	Confirmation	
Willingness to Pay	3.60 € ***^a^	1.73 € **^b^
Poe test	Negative Disconfirmation	
CONV (Purchase Intention, %)	24.6% ^x^	21.8% ^x^
ANOVA	Confirmation	
CONV (Willingness to Pay)	2.72 € ***^b^	2.60 € ***^b^
Poe test	Confirmation	
PREM (Purchase Intention, %)	19.3% ^x^	14.9% ^y^
ANOVA	Negative Disconfirmation	
PREM (Willingness to Pay)	3.57 € ***^a^	2.69 € ***^b^
Poe test	Negative Disconfirmation	
NONE (% selected)	12.0% ^x^	12.4% ^x^
ANOVA	Confirmation	

Within each case-study, products with different superscript letters in rows (x,y) differ (*p* < 0.05). a, b, refer to the difference across products by column at 95% confidence interval. *** *p* < 0.01.

**Table 5:** The principal component analysis (PCA) and individual FNS score after dropping items 8 and 9.

The FNS Items	F1	F2
Item 1 I am constantly sampling new and different foods	0.76	−0.20
Item 4 I like foods from different cultures	0.88	−0.18
Item 6 At dinner parties, I will try new foods	0.75	−0.15
Item 10 I like to try ethnic restaurants	0.86	−0.21
Item 2 I do not trust new foods	−0.28	0.77
Item 3 If I don’t know what a food is, I will not try it	0.04	0.78
Item 5 Ethnic food looks weird to eat	−0.47	0.59
Item 7 I am afraid to eat things I have never had before	−030	0.73
Explained variance (%)	38.4	27.6
Total Explained variance (%)	66.0	
KMO Test	0.794	
Bartlett Test (significance)	441.6 (0.000)	
NFS score	27.68	
Std. Deviation	10.56	
Min.	8.00	
Max.	72.00	

**Table 6:** Results of the two-steps cluster analyses using the adapted FNS.

Cluster 1 Low FN C1	Size (%)	20.0%
	Consumers number	24
	FNS score	13.67
	Standard deviation	2.47
Cluster 2 Average FN C2	Size (%)	34.2%
	Consumers number	41
	FNS score	23.16
	Standard deviation	2.95
Cluster 3 High FN C3	Size (%)	45.8%
	Consumers number	55
	FNS score	36.60
	Standard deviation	6.06

**Table 7:** Food neophobia and the non-hypothetical WTP and PI.

		Expected Before Tasting	Experienced After Tasting
Low FN size		24 consumers	
Purchase Intention	TPP	17.71%	26.56%
	ITPP1	12.50% ^a^	12.50%
	ITPP2	31.77% ^a^	27.60% ^a^
	CONV	18.75%	20.83%
	PREM	15.63%	8.85%
	NONE	3.65% ^b^	4.10% ^b^
WTP	TPP	3.87 € ^a^	4.31 €
	ITPP1	3.60 € ^a^	2.70 €
	ITPP2	4.60 € ^a^	2.34 €
	CONV	2.71 €	2.90 €
	PREM	3.52 €	2.77 €
Average FN size		41 consumers	
Purchase Intention	TPP	16.16%	21.68%
	ITPP1	14.63%	13.27%
	ITPP2	17.07%	17.80%
	CONV	25.30%	15.86%
	PREM	17.68%	21.68%
	NONE	9.15%	9.71%
WTP	TPP	3.71 €	3.38 €
	ITPP1	3.50 €	2.79 €
	ITPP2	3.71 €	1.86 €
	CONV	2.87 €	2.01 €
	PREM	3.54 €	2.89 €
High FN size		55 consumers	
Purchase Intention	TPP	12.27%	20.42%
	ITPP1	7.27% ^b^	11.97%
	ITPP2	14.32% ^b^	16.76% ^b^
	CONV	26.59%	20.42%
	PREM	22.05%	14.93%
	NONE	17.50% ^a^	14.93% ^a^
WTP	TPP	2.88 € ^b^	3.43 €
	ITPP1	2.88 € ^b^	2.41 €
	ITPP2	3.34 € ^b^	1.89 €
	CONV	2.86 €	2.88 €
	PREM	3.68 €	2.19 €

^a,b^: Refer to significant difference between the low and high FN clusters (column comparison) for the analysed products. Differences between clusters were highlighted by shadowed cells.

**Table 8:** The FN associations with the expected liking.

	Low FN	Average FN	High FN
Probabilistic expected liking (TPP)	79.2 ^a^	70.4	64.4 ^b^
Informed liking (TPP)	7.6 ^a^	7.1	6.8 ^b^
Probabilistic expected liking (ITPP1)	74.5 ^a^	66.0	59.7 ^b^
Informed liking (ITPP1)	5.3 ^a^	5.6	5.4 ^a^
Probabilistic expected liking (ITPP2)	80.5 ^a^	65.5	63.4 ^b^
Informed liking (ITPP2)	6.5 ^a^	5.7	5.4 ^a^
Probabilistic expected liking (CONV)	81.0 ^a^	69.7	70.8 ^a^
Informed liking (CONV)	6.7 ^a^	6.5	6.4 ^a^
Probabilistic expected liking (PREM)	82.9 ^a^	79.1	71.2 ^a^
Informed liking (PREM)	6.6 ^a^	6.0	6.6 ^a^

^a,b^ Denotes significant difference at 95% between clusters (shadowed cells).

## Supplementary Materials

The following are available online at https://www.mdpi.com/2072-6643/11/2/444/s1.
