# Supplementary material for: Health Innovation in Patty Products. The Role of Food Neophobia in Consumers’ Non-Hypothetical Willingness to Pay, Purchase Intention and Hedonic Evaluation
Source: Nutrients. 2019 Feb 20;11(2):444. doi: 10.3390/nu11020444 (PMC6412754; doi:10.3390/nu11020444)
Supplement: Supplementary file 1 [file nutrients-11-00444-s001.pdf]

CONSENT FORM

This activity in which you will participate is a part of a European research project called TREASURE (www.treasure.kis.si). This project is an academic research and does not represent any commercial or company interest.

In the development of this project we will study the preferences of European consumers towards different types of products made from different traditional and untapped pig breeds that are raised in different European countries.

In our case study, the _______________________ will analyse the consumers' preferences in ___________towards the consumption and purchasing behaviour of _______________

The session will last about one and half hour. It will consist of different steps with questionnaires and a sensory test of _________________. More details are given in the next explanation.

The information requested in this experiment will be **used exclusively for research and confidentiality is absolutely guaranteed**. It is not necessary to give us information you do not want to provide.

Thanks in advance for participating to our research

DATE: ______________________2017

Signature:

Funded by European Union
Horizon 2020
Grant agreement No 634476

© *TREASURE*

Questionnaire number:_________________ Date:_________________________

The information requested will be used <u>exclusively for research</u> and its confidentiality is guaranteed. It is not necessary to give us information that you don't need to do. Thank you in advance for your contribution to our investigation.

The shadowed cells indicate where to put your specific product or value according to each case study.

1. Approximately, how many times a <u>week</u> do you eat pork products (fresh/processed)?____times a week

2. Approximately, how many time a <u>month</u>/week you consume………..?: ________times a month/week

3. When was the last time you eat …………..at home?__________days ago

☐ One week ago ☐ 2 weeks ago ☐ more than 2 weeks ago ☐ I don't remember

4. Do you have ……….. at home? ☐ Yes ☐ No ☐ I don't know

5. Where do you usually purchase your pork products? (Choose one: the regular place)

☐ Butcher (not of supermarket) ☐ Traditional market

☐ Supermarket/Hypermarket ☐ Other place: _________________

6. Taking into consideration that the average expenditure on food of households in by month …Country/region……….. during …year….. was ……….€/household, approximately, can you please sort your expenditure on food and non-alcoholic beverages of your household according to the average (<u>excluding restaurants</u>)?

☐ Far below average ☐ Below average ☐ On average

☐ Above average ☐ Far above average ☐ I don't know

7. Do you plan to purchase ………… within the next seven days?

☐ No ☐ Yes ☐ I don't know

8. Do you remember the price of ……. you purchased last time?

☐ No, I do not remember

☐ Yes, I remember **8.1.** If yes, how much you paid for your most purchased format?

☐ ________ € / Format:_________________

9. Have you heard about the ……… pigs breed?

☐ No ☐ Yes **9.1** if yes, how you heard about? _________________________

____

10. Have you seen before the ………….. breed?

☐ No ☐ Yes **10.1** if yes, Where did you see it?

☐ On TV ☐ Internet ☐ At farm ☐ Other:__________

11. Have you know some of the products of the ……… pigs breed?

☐ No ☐ Yes **11.1** if yes, which products do you know?

____

12. Have you tasted/eaten any pork product from this breed?

☐ No ☐ Yes 12.1 if yes, which products do you eat? _________________________

____

13. Please state your degree of agreement or disagreement with the following **AFFIRMATIONS**?

13.1. I am constantly tasting /consuming new food?								
Disagree Very Strongly	Disagree Strongly	Disagree Moderately	Disagree Slightly	Neutral	Agree Slightly	Agree Moderately	Agree Strongly	Agree very Strongly
1	2	3	4	5	6	7	8	9

13.2. I don't trust new foods								
Disagree Very Strongly	Disagree Strongly	Disagree Moderately	Disagree Slightly	Neutral	Agree Slightly	Agree Moderately	Agree Strongly	Agree very Strongly
1	2	3	4	5	6	7	8	9

13.3. If I don't know what a food is, I won't try it								
Disagree Very Strongly	Disagree Strongly	Disagree Moderately	Disagree Slightly	Neutral	Agree Slightly	Agree Moderately	Agree Strongly	Agree very Strongly
1	2	3	4	5	6	7	8	9

13.4. I like foods from different cultures								
Disagree Very Strongly	Disagree Strongly	Disagree Moderately	Disagree Slightly	Neutral	Agree Slightly	Agree Moderately	Agree Strongly	Agree very Strongly
1	2	3	4	5	6	7	8	9

13.5. Ethnic food looks weird to eat								
Disagree Very Strongly	Disagree Strongly	Disagree Moderately	Disagree Slightly	Neutral	Agree Slightly	Agree Moderately	Agree Strongly	Agree very Strongly
1	2	3	4	5	6	7	8	9

13.6. At dinner parties with food, I try new foods								
Disagree Very Strongly	Disagree Strongly	Disagree Moderately	Disagree Slightly	Neutral	Agree Slightly	Agree Moderately	Agree Strongly	Agree very Strongly
1	2	3	4	5	6	7	8	9

13.7. I am afraid to eat things I have never eat before								
Disagree Very Strongly	Disagree Strongly	Disagree Moderately	Disagree Slightly	Neutral	Agree Slightly	Agree Moderately	Agree Strongly	Agree very Strongly
1	2	3	4	5	6	7	8	9

13.8. I am very particular about the foods I eat								
Disagree Very Strongly	Disagree Strongly	Disagree Moderately	Disagree Slightly	Neutral	Agree Slightly	Agree Moderately	Agree Strongly	Agree very Strongly
1	2	3	4	5	6	7	8	9

13.9. I would eat almost anything								
Disagree Very Strongly	Disagree Strongly	Disagree Moderately	Disagree Slightly	Neutral	Agree Slightly	Agree Moderately	Agree Strongly	Agree very Strongly
1	2	3	4	5	6	7	8	9

13.10. I like to try ethnic restaurants								
Disagree Very Strongly	Disagree Strongly	Disagree Moderately	Disagree Slightly	Neutral	Agree Slightly	Agree Moderately	Agree Strongly	Agree very Strongly
1	2	3	4	5	6	7	8	9

14. On a scale from 1 to 9, please indicate your level of agreement or disagreement with **WHAT YOU UNDERSTAND BY TRADITIONAL PORK PRODUCTS**?

**14.1. Are anchored to the past**								
Disagree Very Strongly	Disagree Strongly	Disagree Moderately	Disagree Slightly	Neutral	Agree Slightly	Agree Moderately	Agree Strongly	Agree very Strongly
1	2	3	4	5	6	7	8	9

**14.2. Are tied to specific localities, regions or countries**								
Disagree Very Strongly	Disagree Strongly	Disagree Moderately	Disagree Slightly	Neutral	Agree Slightly	Agree Moderately	Agree Strongly	Agree very Strongly
1	2	3	4	5	6	7	8	9

**14.3. Evoke strong memories of childhood**								
Disagree Very Strongly	Disagree Strongly	Disagree Moderately	Disagree Slightly	Neutral	Agree Slightly	Agree Moderately	Agree Strongly	Agree very Strongly
1	2	3	4	5	6	7	8	9

**14.4. Are frequently consumed products**								
Disagree Very Strongly	Disagree Strongly	Disagree Moderately	Disagree Slightly	Neutral	Agree Slightly	Agree Moderately	Agree Strongly	Agree very Strongly
1	2	3	4	5	6	7	8	9

**14.5. Are associated to specific celebrations and/or seasons**								
Disagree Very Strongly	Disagree Strongly	Disagree Moderately	Disagree Slightly	Neutral	Agree Slightly	Agree Moderately	Agree Strongly	Agree very Strongly
1	2	3	4	5	6	7	8	9

**14.6. Are produced following recipes passed from generation to generation**								
Disagree Very Strongly	Disagree Strongly	Disagree Moderately	Disagree Slightly	Neutral	Agree Slightly	Agree Moderately	Agree Strongly	Agree very Strongly
1	2	3	4	5	6	7	8	9

**14.7. Are produced in a domestic setting or by artisans**								
Disagree Very Strongly	Disagree Strongly	Disagree Moderately	Disagree Slightly	Neutral	Agree Slightly	Agree Moderately	Agree Strongly	Agree very Strongly
1	2	3	4	5	6	7	8	9

**14.8. Helps local economies**								
Disagree Very Strongly	Disagree Strongly	Disagree Moderately	Disagree Slightly	Neutral	Agree Slightly	Agree Moderately	Agree Strongly	Agree very Strongly
1	2	3	4	5	6	7	8	9

**14.9. Are environmental friendly**								
Disagree Very Strongly	Disagree Strongly	Disagree Moderately	Disagree Slightly	Neutral	Agree Slightly	Agree Moderately	Agree Strongly	Agree very Strongly
1	2	3	4	5	6	7	8	9

**14.10. Possess distinctive and positive sensory merits**								
Disagree Very Strongly	Disagree Strongly	Disagree Moderately	Disagree Slightly	Neutral	Agree Slightly	Agree Moderately	Agree Strongly	Agree very Strongly
1	2	3	4	5	6	7	8	9

**14.11. Are of low quality**								
Disagree Very Strongly	Disagree Strongly	Disagree Moderately	Disagree Slightly	Neutral	Agree Slightly	Agree Moderately	Agree Strongly	Agree very Strongly
1	2	3	4	5	6	7	8	9

14.12. Are safe								
Disagree Very Strongly	Disagree Strongly	Disagree Moderately	Disagree Slightly	Neutral	Agree Slightly	Agree Moderately	Agree Strongly	Agree very Strongly
1	2	3	4	5	6	7	8	9

14.13. Are authentic and genuine products								
Disagree Very Strongly	Disagree Strongly	Disagree Moderately	Disagree Slightly	Neutral	Agree Slightly	Agree Moderately	Agree Strongly	Agree very Strongly
1	2	3	4	5	6	7	8	9

14.14. Are a part of an area's gastronomic heritage								
Disagree Very Strongly	Disagree Strongly	Disagree Moderately	Disagree Slightly	Neutral	Agree Slightly	Agree Moderately	Agree Strongly	Agree very Strongly
1	2	3	4	5	6	7	8	9

14.15. May help to maintain natural landscape								
Disagree Very Strongly	Disagree Strongly	Disagree Moderately	Disagree Slightly	Neutral	Agree Slightly	Agree Moderately	Agree Strongly	Agree very Strongly
1	2	3	4	5	6	7	8	9

14.16. Are expensive products								
Disagree Very Strongly	Disagree Strongly	Disagree Moderately	Disagree Slightly	Neutral	Agree Slightly	Agree Moderately	Agree Strongly	Agree very Strongly
1	2	3	4	5	6	7	8	9

14.17. Are not healthy and contains higher fat amount								
Disagree Very Strongly	Disagree Strongly	Disagree Moderately	Disagree Slightly	Neutral	Agree Slightly	Agree Moderately	Agree Strongly	Agree very Strongly
1	2	3	4	5	6	7	8	9

14.18. Has narrow assortment of varieties and flavours								
Disagree Very Strongly	Disagree Strongly	Disagree Moderately	Disagree Slightly	Neutral	Agree Slightly	Agree Moderately	Agree Strongly	Agree very Strongly
1	2	3	4	5	6	7	8	9

14.19. Are available at the point of purchase								
Disagree Very Strongly	Disagree Strongly	Disagree Moderately	Disagree Slightly	Neutral	Agree Slightly	Agree Moderately	Agree Strongly	Agree very Strongly
1	2	3	4	5	6	7	8	9

14.20. Are time consuming when prepared								
Disagree Very Strongly	Disagree Strongly	Disagree Moderately	Disagree Slightly	Neutral	Agree Slightly	Agree Moderately	Agree Strongly	Agree very Strongly
1	2	3	4	5	6	7	8	9

14.21. Has unattractive appearance								
Disagree Very Strongly	Disagree Strongly	Disagree Moderately	Disagree Slightly	Neutral	Agree Slightly	Agree Moderately	Agree Strongly	Agree very Strongly
1	2	3	4	5	6	7	8	9

14.22. Are difficult to prepare								
Disagree Very Strongly	Disagree Strongly	Disagree Moderately	Disagree Slightly	Neutral	Agree Slightly	Agree Moderately	Agree Strongly	Agree very Strongly
1	2	3	4	5	6	7	8	9

15. Please mention 2 products that you consider as traditional pork products?

Product 1: ____

Product 2: ____

16. Residence:_________________ (write the name of the place)

17. Have you lived in a rural area? ☐ No ☐ Yes

18. Birth year:____________(put the year)

19. Gender: ☐ Women ☐ Men

20. How many members are you at home (including yourself):_____Members (put number)

21. Do you have children less than 12 years old in your household? ☐ No ☐ Yes

21.1. If yes, how many?:____________

22. What is your education level?

☐ Not completed elementary studies ☐ Elementary studies

☐ Secondary studies ☐ University studies

23. Taking into consideration that the monthly average net income of households in ………….. is ……….€/household, can you please sort the monthly net income of your household according to the average?

☐ Far below average ☐ Below average ☐ Average

☐ Above average ☐ Far above average ☐ I don't know

24. Occupation: ☐ Student

☐ Employee

☐ Self-Business

☐ Retired

☐ Housewife

☐ Unemployed

☐ Other:____________

25. Profession (if applied):________________________

CHOICE EXPERIMENT

- In this experiment you will find DIFFERENT CARDS that represent different PURCHASING MOMENTS of ______________ for CONSUMPTION OF_____________ AT HOME that should be answered before and after a sensory test.
- Each card contains different types of _______________ that are the SAME ON ALL CARDS but with DIFFERENT PRICES
- In the DIFFERENT CARDS, you must answer SEVERAL QUESTIONS about your PREFERENCES for the ______________ and if you WOULD PURCHASE IT for present or future consumption.
- This is a REAL EXPERIMENT in which at the END of answering the questions in ALL CARDS (before and after sensory test), we will carry out a real PURCHASING ACTIVITY of the __________________ by using the MONEY we GAVE you at the beginning of the experiment:
  - ✓ From the different cards, we will RANDOMLY choose ONLY ONE ___product____________ from all cards. Later we will check what WAS YOUR PREFERENCE FOR THE SELECTED PRODUCT and what was your answer for the question "if you WOULD PURCHASE IT".
  - ✓ If you answered YES YOU WOULD PURCHASE IT, then you should PAY its POSTED PRICE on the label and TAKE THE SELECTED PRODUCT using the money we gave you at the beginning of the experiment. ALL the PRODUCTS presented in the cards are physically available. So, at the end of the experiment, you will GET your selected product to take it home.
- So it is IMPORTANT to THINK CAREFULLY what you REALLY PREFER and if you WOULD PURCHASE IT in a real purchase situation.
- ¿WHAT ARE the DIFFERENT TYPE of ____________ presented in the cards? How should be answered? Let's go for the first CARD for their DESCRIPTION.

CHOICE CARDS HERE

1. You'll find a set of total number cards. In each card you are asked to answer the corresponding questions

CARD # 1 (Before the hedonic Evaluation)

PRODUCT A	PRODUCT B	PRODUCT C	PRODUCT D	PRODUCT E
PRICE Q	PRICE T	PRICE Z	PRICE V	PRICE W

Would you purchase any of the presented products ☐ Yes ☐ No

Which product is the MOST PREFERRED that you would purchase for sure? ☐ A ☐ B ☐ C ☐ D ☐ E

CARD # 1 (Before the hedonic Evaluation)

PRODUCT A	PRODUCT B	PRODUCT C	PRODUCT D	PRODUCT E
PRICE Q	PRICE T	PRICE Z	PRICE V	PRICE W

Would you purchase any of the presented products ☐ Yes ☐ No

Which product is the MOST PREFERRED that you would purchase for sure? ☐ A ☐ B ☐ C ☐ D ☐ E

When all cards are answered, the following-up questions are presented.

ELECTION # 1

26. Describe how difficult it was your selection of the products?

Absolutely EASY	Highly EASY	Moderately EASY	Slightly EASY	Neutral	Slightly DIFFICULT	Moderately DIFFICULT	Highly DIFFICULT	Absolutely DIFFICULT
1	2	3	4	5	6	7	8	9

27. To which degree were you sure in selecting the products?

Absolutely SURE	Highly SURE	Moderately SURE	Slightly SURE	Neutral	Slightly UNSURE	Moderately UNSURE	Highly UNSURE	Absolutely UNSURE
1	2	3	4	5	6	7	8	9

ELECTION # 2

28. Describe how difficult it was your selection of the products?

Absolutely EASY	Highly EASY	Moderately EASY	Slightly EASY	Neutral	Slightly DIFFICULT	Moderately DIFFICULT	Highly DIFFICULT	Absolutely DIFFICULT
1	2	3	4	5	6	7	8	9

29. To which degree were you sure in selecting the products?

Absolutely SURE	Highly SURE	Moderately SURE	Slightly SURE	Neutral	Slightly UNSURE	Moderately UNSURE	Highly UNSURE	Absolutely UNSURE
1	2	3	4	5	6	7	8	9

THE LAST 2 QUESTIONS ON PROBABILITIES
(using the label of each specific Choice)

30. In your opinion, what is the PROBABILITY that you WOULD LIKE these PRODUCTS? For example, a 0% probability would mean there is NO chance that you like the product; whereas, a 100% probability would mean that you will like the product for certain.

- ☐ There is a _______% of probability that I will like the Product A
- ☐ There is a _______% of probability that I will like the Product B
- ☐ There is a _______% of probability that I will like the Product C
- ☐ There is a _______% of probability that I will like the Product D
- ☐ There is a _______% of probability that I will like the Product E

31. In your opinion, what is the PROBABILITY that these PRODUCTS WOULD BE healthy/with higher quality? For example, a 0% probability would mean there is NO chance that the product WOULD BE healthy/higher quality; whereas, a 100% probability would mean that the product WOULD BE healthy/higher quality for certain

- ☐ There is a _______% of probability that the Product A will be healthy or with high quality (Depending on each case study)
- ☐ There is a _______% of probability that the Product B will be healthy or with high quality(Depending on each case study)
- ☐ There is a _______% of probability that the Product C will be healthy or with high quality(Depending on each case study)
- ☐ There is a _______% of probability that the Product D will be healthy or with high quality(Depending on each case study)
- ☐ There is a _______% of probability that the Product E will be healthy or with high quality(Depending on each case study)

Consumer number____ Date_____________

You'll receive five coded samples in a given order. Please, taste every sample one by one, taking some toasted bread and drinking some water between one and another. For every sample tasted, please indicate how much you liked the product by putting a cross in the square corresponding to your assessment, from "dislike extremely" to "like extremely".

	SAMPLE CODE	DISLIKE EXTREMELY	DISLIKE VERY MUCH	DISLIKE MODERATELY	DISLIKE SLIGHTLY	NOR LIKE OR DISLIKE	LIKE SLIGHTLY	LIKE MODERATELY	LIKE VERY MUCH	LIKE EXTREMELY
**Sample 1**										
**Sample 2**										
**Sample 3**										
**Sample 4**										
**Sample 5**										

Consumer number____ Date___________

Here you can see five labels corresponding to five different products. For each one of them you'll have to indicate how much you think you'd like it by putting a cross corresponding to your assessment within the scale below, that goes from "dislike extremely" to "like extremely". For each assessment we ask you to consider **only the information reported in the product label** (you won't receive any sample for this part of the test).

**Product A**	
Icon of untapped pig	Name of the untapped pig and product
Description	

**Product B**	
Icon of untapped pig	Name of the untapped pig and product
Description	

**Product C**	
Icon of untapped pig	Name of the untapped pig and product
Description	

**Product D**	
commercial product	Name of product
Description (regular quality)	

**Product E**	
commerical product	Name of product
Description (Premium quality)	

	DISLIKE EXTREMELY	DISLIKE VERY MUCH	DISLIKE MODERATELY	DISLIKE SLIGHTLY	NOR LIKE OR DISLIKE	LIKE SLIGHTLY	LIKE MODERATELY	LIKE VERY MUCH	LIKE EXTREMELY
**Product A**									
**Product B**									
**Product C**									
**Product D**									
**Product E**									

Consumer number____ Date___________

As you've done for the first part of the test, please taste another time all the five products in the given order. Please note that now each coded sample received correspond to one of the label reported below. This time you're asked to assess each one of them as you did before, although this time **you have to consider both information on the label and product sensory acceptability** (How much do you like it?). Remember: taste every sample one by one and take some bread and drink some water between one sample and another.

**sample code (product A)**	
Icon of untapped pig	Name of the untapped pig and product
Description	

**sample code (Product B)**	
Icon of untapped pig	Name of the untapped pig and product
Description	

**sample code (Product C)**	
Icon of untapped pig	Name of the untapped pig and product
Description	

**sample code (Product D)**	
commercial product	Name of product
Description (regular quality)	

**sample code (Product E)**	
commerical product	Name of product
Description (Premium quality)	

	**SAMPLE CODE**	**DISLIKE EXTREMELY**	**DISLIKE VERY MUCH**	**DISLIKE MODERATELY**	**DISLIKE SLIGHTLY**	**NOR LIKE OR DISLIKE**	**LIKE SLIGHTLY**	**LIKE MODERATELY**	**LIKE VERY MUCH**	**LIKE EXTREMELY**
**Sample 1**										
**Sample 2**										
**Sample 3**										
**Sample 4**										
**Sample 5**										

FORMULARIO DE CONSENTIMIENTO

Esta actividad en la que usted va a participar se enmarca dentro del proyecto europeo de investigación **TREASURE** (www.treasure.kis.si). Este proyecto no representa ningún interés comercial y tampoco está relacionado con ninguna empresa.

En el desarrollo de este proyecto estudiaremos las preferencias de los consumidores europeos hacia diferentes tipos de productos elaborados a partir de diferentes razas de porcino tradicionales y autóctonas que se crían en diferentes países europeos.

En nuestro caso, el CREDA (Centre de Recerca en Economia i Desenvolupament Agroalimentari) de la Universidad Politécnica de Catalunya y el IRTA (Institut de Recerca i Tecnologia Agroalimentàries) analizaremos las preferencias hacia el consumo de hamburguesas.

La sesión durará aproximadamente una hora y media y consta de un cuestionario en diferentes etapas y una prueba sensorial de hamburguesas, tal y como les iremos informando a continuación. La información solicitada en este experimento **será utilizada exclusivamente para la investigación y su confidencialidad está absolutamente garantizada**. No es necesario que nos dé alguna información que usted no quiera dar.

Gracias de antemano por su colaboración a nuestra investigación

FECHA: Barcelona a _________________ de 2017

FIRMA:

Funded by European Union
Horizon 2020
Grant agreement No 634476

© *TREASURE*

NÚMERO ENQUESTAT:___________ **DATA:________________________**

La informació sol·licitada serà utilitzada exclusivament per a la recerca i la seva confidencialitat està garantida. No és necessari que ens doni cap informació que vostè no vulgui donar. Gràcies per endavant per la seva col·laboració a la nostra recerca.

1. Quantes vegades **APROXIMADAMENT** a la **SETMANA** consumeix carn de porc (fresca i/o processada)?_____ Vegades/setmana

2. Quantes vegades **APROXIMADAMENT** al **MES** consumeix hamburgueses (porc, vedella, mixtes) a casa? ______ Vegades/mes (s'exclouen les hamburgueses de pollastre, gall dindi, peix, conill i altres carns)

3. Quan va ser **l'ÚLTIMA** vegada que **VA CONSUMIR** hamburguesa (porc, vedella, mixtes) a casa?
   ☐Fa ________dies ☐La setmana passada ☐Fa 2 setmanes
   ☐Fa més de 2 setmanes ☐No me'n recordo ☐Altres:____________

4. **TÉ** hamburgueses (porc, vedella, mixtes) a **CASA**? ☐No ☐Sí ☐ No ho sé

5. On **COMPRA HABITUALMENT** hamburgueses (porc, vedella, mixtes)? (**NOMÉS 1** opció)
   ☐Carnisseria (no del supermercat) ☐Mercats tradicionals (parades)
   ☐Supermercat/hipermercat ☐Altres: _______________________

6. Tenint en compte que la **DESPESA MITJANA MENSUAL** per **LLAR** en **ALIMENTACIÓ** a Catalunya és de 360 €/llar (incloent les begudes no alcohòliques), en quina posició està la despesa mitjana mensual de la seva llar respecte a la mitjana (excloent els restaurants)?
   ☐Molt per sota la mitjana ☐Per sota de la mitjana ☐Igual a la mitjana
   ☐Per sobre de la mitjana ☐Molt per sobre de la mitjana ☐No ho sé

7. Té previst comprar hamburgueses (porc, vedella, mixtes) en els propers **SET DIES**?
   ☐No ☐Sí ☐No ho sé

8. Se'n **RECORDA** del **PREU** de les hamburgueses (porc, vedella, mixtes) que **VA COMPRAR** l'última vegada? ☐No, no me'n recordo ☐Sí me'n recordo

   8.1. En cas **AFIRMATIU**, Quin va ser el **PREU** que **VA PAGAR** pel format d'hamburgueses que va comprar? Contesti **NOMÉS** el **FORMAT** que més compra **HABITUALMENT**
   ☐________ € per Kg d'hamburgueses
   ☐________ € per unitat d'hamburguesa
   ☐________ € per safata de 2 hamburgueses
   ☐________ € per safata de 4 hamburgueses
   ☐________ € per un altre format:_________________________________

9. Ha sentit parlar del ***Porc Negre Mallorquí***?
   ☐No ☐Sí **9.1** En cas **AFIRMATIU**, On n'ha sentit parlar?
   ____

10. Ha vist alguna vegada el ***Porc Negre Mallorquí***?
    ☐No ☐Sí **10.1** En cas **AFIRMATIU**, On l'ha vist? A:
    ☐La televisió ☐Internet ☐La granja ☐Altres:____________

11. Coneix algun producte del ***Porc Negre Mallorquí***?
    ☐No ☐Si **11.1** En cas **AFIRMATIU**, Quins productes coneix?
    ____
    ____

12. Ha provat/consumit alguna vegada algun producte del ***Porc Negre Mallorquí***?
    ☐No ☐Sí **12.1** En cas **AFIRMATIU**, Quins productes ha provat/consumit? _
    ____

13. En una escala d'1 a 9, Indiqui el seu grau **D'ACORD** o **DESACORD** amb les següents **AFIRMACIONS**?

**13.1 Estic constantment provant aliments nous i diferents**								
Absolutament en desacord	Molt en desacord	Moderadament en desacord	Poc en desacord	Neutral	Poc d'acord	Moderadament d'acord	Molt d'acord	Absolutament d'acord
**1**	**2**	**3**	**4**	**5**	**6**	**7**	**8**	**9**

**13.2 No confio en els aliments nous**								
Absolutament en desacord	Molt en desacord	Moderadament en desacord	Poc en desacord	Neutral	Poc d'acord	Moderadament d'acord	Molt d'acord	Absolutament d'acord
**1**	**2**	**3**	**4**	**5**	**6**	**7**	**8**	**9**

**13.3 Si no conec què hi ha en un aliment, no ho provo**								
Absolutament en desacord	Molt en desacord	Moderadament en desacord	Poc en desacord	Neutral	Poc d'acord	Moderadament d'acord	Molt d'acord	Absolutament d'acord
**1**	**2**	**3**	**4**	**5**	**6**	**7**	**8**	**9**

**13.4 M'agraden els menjars de països diferents**								
Absolutament en desacord	Molt en desacord	Moderadament en desacord	Poc en desacord	Neutral	Poc d'acord	Moderadament d'acord	Molt d'acord	Absolutament d'acord
**1**	**2**	**3**	**4**	**5**	**6**	**7**	**8**	**9**

**13.5 El menjar ètnic em sembla massa estrany per menjar**								
Absolutament en desacord	Molt en desacord	Moderadament en desacord	Poc en desacord	Neutral	Poc d'acord	Moderadament d'acord	Molt d'acord	Absolutament d'acord
**1**	**2**	**3**	**4**	**5**	**6**	**7**	**8**	**9**

**13.6 En festes on hi ha menjar, provo nous aliments**								
Absolutament en desacord	Molt en desacord	Moderadament en desacord	Poc en desacord	Neutral	Poc d'acord	Moderadament d'acord	Molt d'acord	Absolutament d'acord
**1**	**2**	**3**	**4**	**5**	**6**	**7**	**8**	**9**

**13.7 Em fa por provar aliments que mai no he provat abans**								
Absolutament en desacord	Molt en desacord	Moderadament en desacord	Poc en desacord	Neutral	Poc d'acord	Moderadament d'acord	Molt d'acord	Absolutament d'acord
**1**	**2**	**3**	**4**	**5**	**6**	**7**	**8**	**9**

**13.8 Sóc molt especial amb els aliments que menjo**								
Absolutament en desacord	Molt en desacord	Moderadament en desacord	Poc en desacord	Neutral	Poc d'acord	Moderadament d'acord	Molt d'acord	Absolutament d'acord
**1**	**2**	**3**	**4**	**5**	**6**	**7**	**8**	**9**

**13.9 Menjaria gairebé de tot**								
Absolutament en desacord	Molt en desacord	Moderadament en desacord	Poc en desacord	Neutral	Poc d'acord	Moderadament d'acord	Molt d'acord	Absolutament d'acord
**1**	**2**	**3**	**4**	**5**	**6**	**7**	**8**	**9**

**13.10 M'agrada provar nous restaurants ètnics**								
Absolutament en desacord	Molt en desacord	Moderadament en desacord	Poc en desacord	Neutral	Poc d'acord	Moderadament d'acord	Molt d'acord	Absolutament d'acord
**1**	**2**	**3**	**4**	**5**	**6**	**7**	**8**	**9**

14. En una escala d’1 a 9, indiqui per favor el seu grau d'acord o desacord amb **EL QUE ENTÉN VOSTÈ PER PRODUCTES TRADICIONALS DE PORC**?.

**14.1 Són productes que estan ancorats al passat**								
Absolutament en desacord	Molt en desacord	Moderadament en desacord	Poc en desacord	Neutral	Poc d'acord	Moderadament d'acord	Molt d'acord	Absolutament d'acord
**1**	**2**	**3**	**4**	**5**	**6**	**7**	**8**	**9**

**14.2 Són productes que estan vinculats a determinades localitats, regions o països**								
Absolutament en desacord	Molt en desacord	Moderadament en desacord	Poc en desacord	Neutral	Poc d'acord	Moderadament d'acord	Molt d'acord	Absolutament d'acord
**1**	**2**	**3**	**4**	**5**	**6**	**7**	**8**	**9**

**14.3 Són productes que evoquen forts records de la infància**								
Absolutament en desacord	Molt en desacord	Moderadament en desacord	Poc en desacord	Neutral	Poc d'acord	Moderadament d'acord	Molt d'acord	Absolutament d'acord
**1**	**2**	**3**	**4**	**5**	**6**	**7**	**8**	**9**

**14.4 Són productes de consum habitual**								
Absolutament en desacord	Molt en desacord	Moderadament en desacord	Poc en desacord	Neutral	Poc d'acord	Moderadament d'acord	Molt d'acord	Absolutament d'acord
**1**	**2**	**3**	**4**	**5**	**6**	**7**	**8**	**9**

**14.5 Són productes que estan associats a celebracions, ocasions i/o temporades específiques**								
Absolutament en desacord	Molt en desacord	Moderadament en desacord	Poc en desacord	Neutral	Poc d'acord	Moderadament d'acord	Molt d'acord	Absolutament d'acord
**1**	**2**	**3**	**4**	**5**	**6**	**7**	**8**	**9**

**14.6 Són productes produïts seguint receptes que han passat de generació en generació**								
Absolutament en desacord	Molt en desacord	Moderadament en desacord	Poc en desacord	Neutral	Poc d'acord	Moderadament d'acord	Molt d'acord	Absolutament d'acord
**1**	**2**	**3**	**4**	**5**	**6**	**7**	**8**	**9**

**14.7 Són productes produïts en un entorn domèstic o per artesans**								
Absolutament en desacord	Molt en desacord	Moderadament en desacord	Poc en desacord	Neutral	Poc d'acord	Moderadament d'acord	Molt d'acord	Absolutament d'acord
**1**	**2**	**3**	**4**	**5**	**6**	**7**	**8**	**9**

**14.8 Són productes que ajuden a les economies locals**								
Absolutament en desacord	Molt en desacord	Moderadament en desacord	Poc en desacord	Neutral	Poc d'acord	Moderadament d'acord	Molt d'acord	Absolutament d'acord
**1**	**2**	**3**	**4**	**5**	**6**	**7**	**8**	**9**

**14.9 Són respectuosos amb el medi ambient**								
Absolutament en desacord	Molt en desacord	Moderadament en desacord	Poc en desacord	Neutral	Poc d'acord	Moderadament d'acord	Molt d'acord	Absolutament d'acord
**1**	**2**	**3**	**4**	**5**	**6**	**7**	**8**	**9**

**14.10 Són productes que tenen mèrits sensorials distintius i positius**								
Absolutament en desacord	Molt en desacord	Moderadament en desacord	Poc en desacord	Neutral	Poc d'acord	Moderadament d'acord	Molt d'acord	Absolutament d'acord
**1**	**2**	**3**	**4**	**5**	**6**	**7**	**8**	**9**

**14.11 Són productes de baixa qualitat**								
Absolutament en desacord	Molt en desacord	Moderadament en desacord	Poc en desacord	Neutral	Poc d'acord	Moderadament d'acord	Molt d'acord	Absolutament d'acord
**1**	**2**	**3**	**4**	**5**	**6**	**7**	**8**	**9**

14.12 Són productes segurs								
Absolutament en desacord	Molt en desacord	Moderadament en desacord	Poc en desacord	Neutral	Poc d'acord	Moderadament d'acord	Molt d'acord	Absolutament d'acord
**1**	**2**	**3**	**4**	**5**	**6**	**7**	**8**	**9**

14.13 Són productes autèntics i genuïns								
Absolutament en desacord	Molt en desacord	Moderadament en desacord	Poc en desacord	Neutral	Poc d'acord	Moderadament d'acord	Molt d'acord	Absolutament d'acord
**1**	**2**	**3**	**4**	**5**	**6**	**7**	**8**	**9**

14.14 Són productes que formen part del patrimoni gastronòmic d'una regió								
Absolutament en desacord	Molt en desacord	Moderadament en desacord	Poc en desacord	Neutral	Poc d'acord	Moderadament d'acord	Molt d'acord	Absolutament d'acord
**1**	**2**	**3**	**4**	**5**	**6**	**7**	**8**	**9**

14.15 Són productes que poden ajudar a mantenir el paisatge natural								
Absolutament en desacord	Molt en desacord	Moderadament en desacord	Poc en desacord	Neutral	Poc d'acord	Moderadament d'acord	Molt d'acord	Absolutament d'acord
**1**	**2**	**3**	**4**	**5**	**6**	**7**	**8**	**9**

14.16 Són productes cars								
Absolutament en desacord	Molt en desacord	Moderadament en desacord	Poc en desacord	Neutral	Poc d'acord	Moderadament d'acord	Molt d'acord	Absolutament d'acord
**1**	**2**	**3**	**4**	**5**	**6**	**7**	**8**	**9**

14.17 Són productes saludables i contenen una quantitat elevada de greix								
Absolutament en desacord	Molt en desacord	Moderadament en desacord	Poc en desacord	Neutral	Poc d'acord	Moderadament d'acord	Molt d'acord	Absolutament d'acord
**1**	**2**	**3**	**4**	**5**	**6**	**7**	**8**	**9**

14.18 Són productes que tenen poc assortit de varietats i sabors								
Absolutament en desacord	Molt en desacord	Moderadament en desacord	Poc en desacord	Neutral	Poc d'acord	Moderadament d'acord	Molt d'acord	Absolutament d'acord
**1**	**2**	**3**	**4**	**5**	**6**	**7**	**8**	**9**

14.19 Són productes que estan disponibles en els punts de venda habituals d'aliments								
Absolutament en desacord	Molt en desacord	Moderadament en desacord	Poc en desacord	Neutral	Poc d'acord	Moderadament d'acord	Molt d'acord	Absolutament d'acord
**1**	**2**	**3**	**4**	**5**	**6**	**7**	**8**	**9**

14.20 Són productes que requereixen molt temps per a la seva elaboració								
Absolutament en desacord	Molt en desacord	Moderadament en desacord	Poc en desacord	Neutral	Poc d'acord	Moderadament d'acord	Molt d'acord	Absolutament d'acord
**1**	**2**	**3**	**4**	**5**	**6**	**7**	**8**	**9**

14.21 Són productes que tenen un aspecte poc atractiu								
Absolutament en desacord	Molt en desacord	Moderadament en desacord	Poc en desacord	Neutral	Poc d'acord	Moderadament d'acord	Molt d'acord	Absolutament d'acord
**1**	**2**	**3**	**4**	**5**	**6**	**7**	**8**	**9**

14.22 Són productes difícils de preparar								
Absolutament en desacord	Molt en desacord	Moderadament en desacord	Poc en desacord	Neutral	Poc d'acord	Moderadament d'acord	Molt d'acord	Absolutament d'acord
**1**	**2**	**3**	**4**	**5**	**6**	**7**	**8**	**9**

15. Indiqui dos productes que considera com a **PRODUCTES TRADICIONALS DE PORC**

☐Producte 1:____

☐Producte 2:____

16. Codi **POSTAL**:_________________

17. Ha viscut alguna vegada en una zona RURAL? ☐No ☐Sí

18. Any de **NAIXEMENT**:____________

19. **GÈNERE**: ☐Dona ☐Home

20. Quantes **PERSONES** hi ha a la seva **LLAR** (inclòs vostè):_________persones

21. Hi ha **MENORS** de **12 ANYS** a la seva llar?

☐No ☐Sí **22.1.** En cas **AFIRMATIU**, quants? ______

22. Nivell d'**ESTUDIS**:

☐Estudis primaris no completats ☐Primaris ☐Secundaris ☐Universitaris

23. Tenint en compte que els INGRESSOS NETS MENSUALS per llar a Catalunya són de 2.500 €/llar, en quina posició estan els ingressos nets mensuals a la seva llar respecte a la mitjana?

☐Molt per sota la mitjana ☐Per sota de la mitjana ☐Igual a la mitjana
☐Per sobre de la mitjana ☐Molt per sobre de la mitjana ☐No ho sé

24. **OCUPACIÓ**: ☐Estudiant ☐Empleat ☐Autònom/empresari
☐Jubilat ☐Mestressa de casa ☐Aturat ☐Altres:

25. **PROFESSIÓ** (si s'escau):________________________

QUADERN DE TARGETES

- A continuació Vostè va a participar en un experiment que té dues tasques: Una PRINCIPAL i altre ESPECIAL. Ambdues tasques es faran ABANS i DESPRÉS de PROVAR diferents tipus d'HAMBURGUESES. La primera està formada per 9 TARGETES (20 minuts) i la segona està formada per 1 TARGETA (3 minuts). Totes les targetes simulen DIFERENTS MOMENTS DE COMPRA d'HAMBURGUESA per al CONSUM EN LA SEVA LLAR.
- CADA TARGETA conté 5 TIPUS D'HAMBURGUESES que es REPETEIXEN en totes les targetes però amb DIFERENTS PREUS que oscil·len de 2 € per una safata de 2 hamburgueses de 250 g a 5.25 € per una safata de 2 hamburgueses de 250 g.
  1. EL primer tipus d'hamburguesa està fet a partir de carn de porc que pertany a la RAÇA AUTÒCTONA DEL PORC NEGRE MALLORQUÍ criada en un sistema extensiu.
  2. EL segon tipus d'hamburguesa està fet a partir de carn de porc que pertany a la RAÇA AUTÒCTONA DEL PORC NEGRE MALLORQUÍ criada en un sistema extensiu. Aquesta hamburguesa està preparada amb una FONT NATURAL DE FIBRA ALIMENTÀRIA que millora les nostres DEFENSES.
  3. EL tercer tipus d'hamburguesa està fet a partir de carn de porc que pertany a la RAÇA AUTÒCTONA DEL PORC NEGRE MALLORQUÍ criada en un sistema extensiu. Aquesta hamburguesa està preparada amb una FONT NATURAL D'ANTIOXIDANTS que ajuda a prevenir malalties CARDIOVASCULARS.
  4. EL quart tipus d'hamburguesa és una hamburguesa MIXTA de PORC i VEDELLA.
  5. El cinquè tipus d'hamburguesa és una hamburguesa de CARN SELECTA de VEDELLA.
- En cada TARGETA, s'ha de CONTESTAR una SÈRIE DE PREGUNTES sobre LES SEVES PREFERÈNCIES respecte dels diferents tipus d'hamburguesa i SI LES COMPRARIA. (SE LI ENSENYARÀ A LA PANTALLA COM CONTESTAR UN EXEMPLE)

- L'experiment és un EXPERIMENT REAL. AL FINAL hi haurà un ACTE REAL DE COMPRA D'HAMBURGUESES amb els DINERS que LI REGALAREM al final de l'experiment. Així, és MOLT IMPORTANT que CONTESTI quines hamburgueses REALMENT PREFEREIX i si les COMPRARIA, ja que les seves respostes implicarien la compra (o no) de les mateixes. Per això, li demanem, per favor, que ACTUÏ COM REALMENT FARIA en un MERCAT i no com li agradaria actuar. L'acte de compra es farà de la següent manera:
  - ✓ PRIMER triem aleatòriament quin dues tasques usem, les d'ABANS o DESPRÉS de provar les hamburgueses.
  - ✓ SEGON triem aleatòriament UNA de les dues TASQUES (la principal o l'especial)
  - ✓ TERCER, si la tasca PRINCIPAL és la triada, seleccionem aleatòriament una TARGETA de totes les TARGETES de la tasca. Si la tasca ESPECIAL és triada, seleccionem la seva ÚNICA TARGETA.
  - ✓ QUART**,** en la targeta triada, si ha contestat CAP de les cinc HAMBURGUESES, rebrà els 10 € íntegrament.
  - ✓ CINQUÈ, en cas contrari i en aquesta mateixa targeta, cada participant triarà aleatòriament un ÚNIC TIPUS d'HAMBURGUESES.
  - ✓ SISÈ, comprovem quin ha estat LA SEVA PREFERÈNCIA i SI COMPRARIA EL TIPUS D'HAMBURGUESA TRIAT. Si ha contestat SÍ LA COMPRARIA, vostè ha de COMPRAR L'HAMBURGUESA TRIADA PAGANT el PREU exposat en la seva etiqueta. En aquest cas, li regalem els 10 € menys el preu de l'hamburguesa triada i la hi lliurarem.
  - ✓ Li DONAREM UN TIQUET per retirar l'hamburguesa triada, indicant-li el lloc i a partir de quin dia.

A

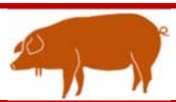

Hamburguesa
Porc Negre Mallorquí

Carn de raça autòctona criada en sistema extensiu

2 Hamburgueses (250 g)
3.00€ **(12.00 €/Kg)**

B

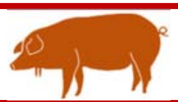

Hamburguesa
Porc Negre Mallorquí

Carn de raça autòctona criada en sistema extensiu
Preparada amb una font natural de fibra alimentària que millora les nostres defenses

2 Hamburgueses (250 g)
4.50€ **(18.00 €/Kg)**

C

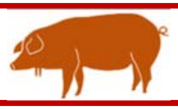

Hamburguesa
Porc Negre Mallorquí

Carn de raça autòctona criada en sistema extensiu
Preparada amb una font natural d'antioxidants que ajuda a prevenir malalties cardiovasculars

2 Hamburgueses (250 g)
3.75€ **(15.00 €/Kg)**

D

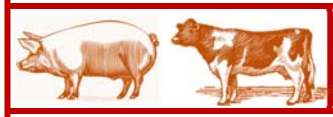

Hamburguesa mixta
Porc i Vedella

2 Hamburgueses (250 g)
2.50€ **(10.00 €/Kg)**

E

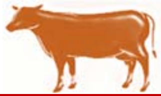

Hamburguesa
Vedella

Carn selecta

2 Hamburgueses (250 g)
5.25€ **(21.00 €/Kg)**

1. Compraria ALGUNA de les CINC hamburgueses? ☐**SI** ☐**NO, CAP de les CINC** TARGETA 1 - ELECCIÓ 1

2. Quina és la seva PRIMERA hamburguesa MÉS PREFERIDA que segur compraria (La MILLOR)?☐**A** ☐**B** ☐**C** ☐**D** ☐**E**

1. Compraria ALGUNA de les CINC hamburgueses? ☐**SI** ☐**NO, CAP de les CINC** TARGETA 1 - ELECCIÓ 2

2. Quina és la seva PRIMERA hamburguesa MÉS PREFERIDA que segur compraria (La MILLOR)?☐**A** ☐**B** ☐**C** ☐**D** ☐**E**

A

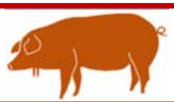

Hamburguesa
Porc Negre Mallorquí

Carn de raça autòctona criada en sistema extensiu

2 Hamburgueses (250 g)
3.75€ **(15.00 €/Kg)**

B

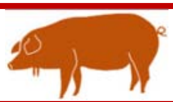

Hamburguesa
Porc Negre Mallorquí

Carn de raça autòctona criada en sistema extensiu
Preparada amb una font natural de fibra alimentària que millora les nostres defenses

2 Hamburgueses (250 g)
5.25€ **(21.00 €/Kg)**

C

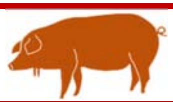

Hamburguesa
Porc Negre Mallorquí

Carn de raça autòctona criada en sistema extensiu
Preparada amb una font natural d'antioxidants que ajuda a prevenir malalties cardiovasculars

2 Hamburgueses (250 g)
4.50€ **(18.00 €/Kg)**

D

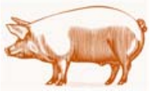

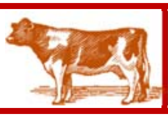

Hamburguesa mixta
Porc i Vedella

2 Hamburgueses (250 g)
2.50€ **(10.00 €/Kg)**

E

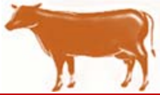

Hamburguesa
Vedella

Carn selecta

2 Hamburgueses (250 g)
3.00€ **(12.00 €/Kg)**

1. Compraria ALGUNA de les CINC hamburgueses? **□SI □NO, CAP de les CINC** TARGETA 2 - ELECCIÓ 1

2. Quina és la seva PRIMERA hamburguesa MÉS PREFERIDA que segur compraria (La MILLOR)?□A □B □C □D □E

1. Compraria ALGUNA de les CINC hamburgueses? **□SI □NO, CAP de les CINC** TARGETA 2 - ELECCIÓ 2

2. Quina és la seva PRIMERA hamburguesa MÉS PREFERIDA que segur compraria (La MILLOR)?□A □B □C □D □E

A

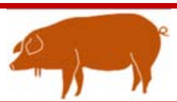

Hamburguesa
Porc Negre Mallorquí

Carn de raça autòctona criada en sistema extensiu

2 Hamburgueses (250 g)
4.50€ **(18.00 €/Kg)**

B

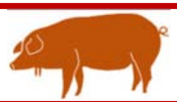

Hamburguesa
Porc Negre Mallorquí

Carn de raça autòctona criada en sistema extensiu
Preparada amb una font natural de fibra alimentària que millora les nostres defenses

2 Hamburgueses (250 g)
3.00€ **(12.00 €/Kg)**

C

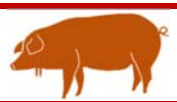

Hamburguesa
Porc Negre Mallorquí

Carn de raça autòctona criada en sistema extensiu
Preparada amb una font natural d'antioxidants que ajuda a prevenir malalties cardiovasculars

2 Hamburgueses (250 g)
4.50€ **(18.00 €/Kg)**

D

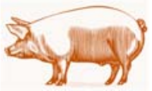

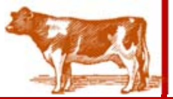

Hamburguesa mixta
Porc i Vedella

2 Hamburgueses (250 g)
3.50€ **(14.00 €/Kg)**

E

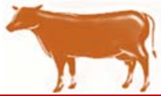

Hamburguesa
Vedella

Carn selecta

2 Hamburgueses (250 g)
4.50€ **(18.00 €/Kg)**

1. Compraria ALGUNA de les CINC hamburgueses? ☐**SI** ☐**NO, CAP de les CINC** TARGETA 3 - ELECCIÓ 1

2. Quina és la seva PRIMERA hamburguesa MÉS PREFERIDA que segur compraria (La MILLOR)?☐**A** ☐**B** ☐**C** ☐**D** ☐**E**

1. Compraria ALGUNA de les CINC hamburgueses? ☐**SI** ☐**NO, CAP de les CINC** TARGETA 3 - ELECCIÓ 2

2. Quina és la seva PRIMERA hamburguesa MÉS PREFERIDA que segur compraria (La MILLOR)?☐**A** ☐**B** ☐**C** ☐**D** ☐**E**

A

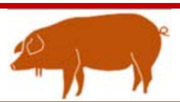

Hamburguesa
Porc Negre Mallorquí

Carn de raça autòctona criada en sistema extensiu

2 Hamburgueses (250 g)
4.50€ **(18.00 €/Kg)**

B

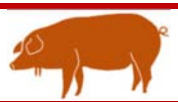

Hamburguesa
Porc Negre Mallorquí

Carn de raça autòctona criada en sistema extensiu
Preparada amb una font natural de fibra alimentària que millora les nostres defenses

2 Hamburgueses (250 g)
5.25€ **(21.00 €/Kg)**

C

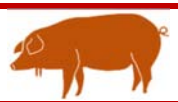

Hamburguesa
Porc Negre Mallorquí

Carn de raça autòctona criada en sistema extensiu
Preparada amb una font natural d'antioxidants que ajuda a prevenir malalties cardiovasculars

2 Hamburgueses (250 g)
3.75€ **(15.00 €/Kg)**

D

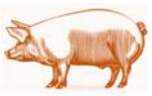

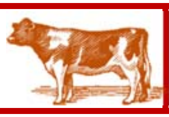

Hamburguesa mixta
Porc i Vedella

2 Hamburgueses (250 g)
3.50€ **(14.00 €/Kg)**

E

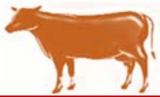

Hamburguesa
Vedella

Carn selecta

2 Hamburgueses (250 g)
3.00€ **(12.00 €/Kg)**

1. Compraria ALGUNA de les CINC hamburgueses? ☐**SI** ☐**NO, CAP de les CINC**

TARGETA 4 - ELECCIÓ 1

2. Quina és la seva PRIMERA hamburguesa MÉS PREFERIDA que segur compraria (La MILLOR)? ☐**A** ☐**B** ☐**C** ☐**D** ☐**E**

1. Compraria ALGUNA de les CINC hamburgueses? ☐**SI** ☐**NO, CAP de les CINC**

TARGETA 4 - ELECCIÓ 2

2. Quina és la seva PRIMERA hamburguesa MÉS PREFERIDA que segur compraria (La MILLOR)? ☐**A** ☐**B** ☐**C** ☐**D** ☐**E**

A

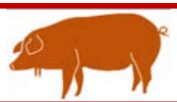

Hamburguesa
Porc Negre Mallorquí

Carn de raça autòctona criada en sistema extensiu

2 Hamburgueses (250 g)
5.25€ **(21.00 €/Kg)**

B

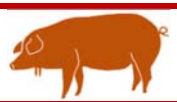

Hamburguesa
Porc Negre Mallorquí

Carn de raça autòctona criada en sistema extensiu
Preparada amb una font natural de fibra alimentària que millora les nostres defenses

2 Hamburgueses (250 g)
3.75€ **(15.00 €/Kg)**

C

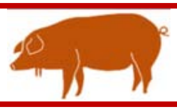

Hamburguesa
Porc Negre Mallorquí

Carn de raça autòctona criada en sistema extensiu
Preparada amb una font natural d'antioxidants que ajuda a prevenir malalties cardiovasculars

2 Hamburgueses (250 g)
3.00€ **(12.00 €/Kg)**

D

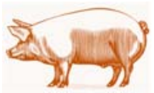
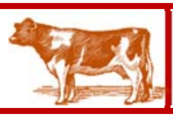

Hamburguesa mixta
Porc i Vedella

2 Hamburgueses (250 g)
3.00€ **(12.00 €/Kg)**

E

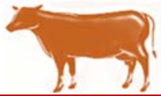

Hamburguesa
Vedella

Carn selecta

2 Hamburgueses (250 g)
4.50€ **(18.00 €/Kg)**

---

1. Compraria ALGUNA de les CINC hamburgueses? ☐**SI** ☐**NO, CAP de les CINC**

TARGETA 5 - ELECCIÓ 1

2. Quina és la seva PRIMERA hamburguesa MÉS PREFERIDA que segur compraria (La MILLOR)? ☐**A** ☐**B** ☐**C** ☐**D** ☐**E**

---

1. Compraria ALGUNA de les CINC hamburgueses? ☐**SI** ☐**NO, CAP de les CINC**

TARGETA 5 - ELECCIÓ 2

2. Quina és la seva PRIMERA hamburguesa MÉS PREFERIDA que segur compraria (La MILLOR)? ☐**A** ☐**B** ☐**C** ☐**D** ☐**E**

A

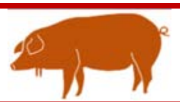

Hamburguesa
Porc Negre Mallorquí

Carn de raça autòctona criada
en sistema extensiu

2 Hamburgueses (250 g)
5.25€ **(21.00 €/Kg)**

B

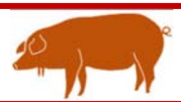

Hamburguesa
Porc Negre Mallorquí

Carn de raça autòctona criada
en sistema extensiu
Preparada amb una font natural de
fibra alimentària que millora
les nostres defenses

2 Hamburgueses (250 g)
3.00€ **(12.00 €/Kg)**

C

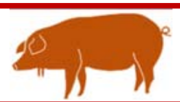

Hamburguesa
Porc Negre Mallorquí

Carn de raça autòctona criada
en sistema extensiu
Preparada amb una font natural
d'antioxidants que ajuda a prevenir
malalties cardiovasculars

2 Hamburgueses (250 g)
3.00€ **(12.00 €/Kg)**

D

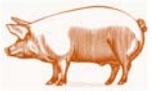

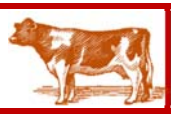

Hamburguesa mixta
Porc i Vedella

2 Hamburgueses (250 g)
3.00€ **(12.00 €/Kg)**

E

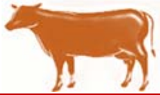

Hamburguesa
Vedella

Carn selecta

2 Hamburgueses (250 g)
5.25€ **(21.00 €/Kg)**

1. Compraria ALGUNA de les CINC hamburgueses? ☐SI ☐NO, CAP de les CINC TARGETA 6 - ELECCIÓ 1

2. Quina és la seva PRIMERA hamburguesa MÉS PREFERIDA que segur compraria (La MILLOR)?☐A ☐B ☐C ☐D ☐E

1. Compraria ALGUNA de les CINC hamburgueses? ☐SI ☐NO, CAP de les CINC TARGETA 6 - ELECCIÓ 2

2. Quina és la seva PRIMERA hamburguesa MÉS PREFERIDA que segur compraria (La MILLOR)?☐A ☐B ☐C ☐D ☐E

A

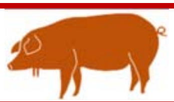

Hamburguesa
Porc Negre Mallorquí

Carn de raça autòctona criada en sistema extensiu

2 Hamburgueses (250 g)
3.00€ **(12.00 €/Kg)**

B

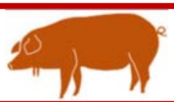

Hamburguesa
Porc Negre Mallorquí

Carn de raça autòctona criada en sistema extensiu
Preparada amb una font natural de fibra alimentària que millora les nostres defenses

2 Hamburgueses (250 g)
4.50€ **(18.00 €/Kg)**

C

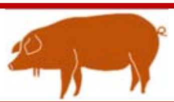

Hamburguesa
Porc Negre Mallorquí

Carn de raça autòctona criada en sistema extensiu
Preparada amb una font natural d'antioxidants que ajuda a prevenir malalties cardiovasculars

2 Hamburgueses (250 g)
5.25€ **(21.00 €/Kg)**

D

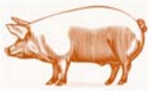

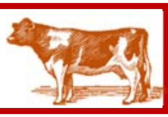

Hamburguesa mixta
Porc i Vedella

2 Hamburgueses (250 g)
2.00€ **(8.00 €/Kg)**

E

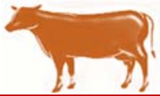

Hamburguesa
Vedella

Carn selecta

2 Hamburgueses (250 g)
3.75€ **(15.00 €/Kg)**

1. Compraria ALGUNA de les CINC hamburgueses? ☐SI ☐NO, CAP de les CINC TARGETA 7 - ELECCIÓ 1

2. Quina és la seva PRIMERA hamburguesa MÉS PREFERIDA que segur compraria (La MILLOR)?☐A ☐B ☐C ☐D ☐E

1. Compraria ALGUNA de les CINC hamburgueses? ☐SI ☐NO, CAP de les CINC TARGETA 7 - ELECCIÓ 2

2. Quina és la seva PRIMERA hamburguesa MÉS PREFERIDA que segur compraria (La MILLOR)?☐A ☐B ☐C ☐D ☐E

A

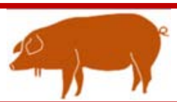

Hamburguesa
Porc Negre Mallorquí

Carn de raça autòctona criada en sistema extensiu

2 Hamburgueses (250 g)
3.75€ **(15.00 €/Kg)**

B

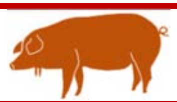

Hamburguesa
Porc Negre Mallorquí

Carn de raça autòctona criada en sistema extensiu
Preparada amb una font natural de fibra alimentària que millora les nostres defenses

2 Hamburgueses (250 g)
3.75€ **(15.00 €/Kg)**

C

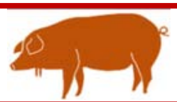

Hamburguesa
Porc Negre Mallorquí

Carn de raça autòctona criada en sistema extensiu
Preparada amb una font natural d'antioxidants que ajuda a prevenir malalties cardiovasculars

2 Hamburgueses (250 g)
5.25€ **(21.00 €/Kg)**

D

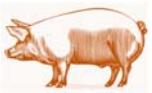
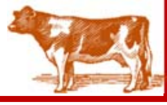

Hamburguesa mixta
Porc i Vedella

2 Hamburgueses (250 g)
2.00€ **(8.00 €/Kg)**

E

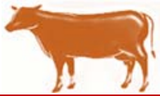

Hamburguesa
Vedella

Carn selecta

2 Hamburgueses (250 g)
3.75€ **(15.00 €/Kg)**

1. Compraria ALGUNA de les CINC hamburgueses? ☐SI ☐NO, CAP de les CINC TARGETA 8 - ELECCIÓ 1

2. Quina és la seva PRIMERA hamburguesa MÉS PREFERIDA que segur compraria (La MILLOR)?☐A ☐B ☐C ☐D ☐E

1. Compraria ALGUNA de les CINC hamburgueses? ☐SI ☐NO, CAP de les CINC TARGETA 8 - ELECCIÓ 2

2. Quina és la seva PRIMERA hamburguesa MÉS PREFERIDA que segur compraria (La MILLOR)?☐A ☐B ☐C ☐D ☐E

E L E C C I Ó 1

26. En una escala d'1 a 9, descrigui com de COMPLICAT li ha estat triar entre els productes de les targetes?

Totalment Fàcil	Molt Fàcil	Lleugerament Fàcil	Poc Fàcil	Neutral	Poc Difícil	Lleugerament Difícil	Molt Difícil	Totalment Difícil
1	2	3	4	5	6	7	8	9

27. En una escala d'1 a 9 indiqui amb quin nivell de CONFIANÇA ha fet la seva elecció dels productes de les targetes?

Totalment Segur	Molt Segur	Lleugerament Segur	Poc Segur	Neutral	Poc Insegur	Lleugerament Insegur	Molt Insegur	Totalment Insegur
1	2	3	4	5	6	7	8	9

E L E C C I Ó 2

28. En una escala d'1 a 9, descrigui com de COMPLICAT li ha estat triar entre els productes de les targetes?

Totalment Fàcil	Molt Fàcil	Lleugerament Fàcil	Poc Fàcil	Neutral	Poc Difícil	Lleugerament Difícil	Molt Difícil	Totalment Difícil
1	2	3	4	5	6	7	8	9

29. En una escala d'1 a 9 indiqui amb quin nivell de CONFIANÇA ha fet la seva elecció dels productes de les targetes?

Totalment Segur	Molt Segur	Lleugerament Segur	Poc Segur	Neutral	Poc Insegur	Lleugerament Insegur	Molt Insegur	Totalment Insegur
1	2	3	4	5	6	7	8	9

A

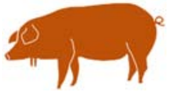

Hamburguesa
Porc Negre Mallorquí

Carn de raça autòctona criada en sistema extensiu

B

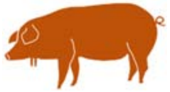

Hamburguesa
Porc Negre Mallorquí

Carn de raça autòctona criada en sistema extensiu
Preparada amb una font natural de fibra alimentària que millora les nostres defenses

C

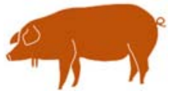

Hamburguesa
Porc Negre Mallorquí

Carn de raça autòctona criada en sistema extensiu
Preparada amb una font natural d'antioxidants que ajuda a prevenir malalties cardiovasculars

D

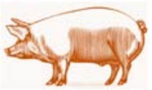

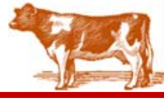

Hamburguesa mixta
Porc i Vedella

E

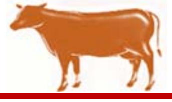

Hamburguesa
Vedella

Carn selecta

30. Segons la seva opinió, amb quina PROBABILITAT CREU QUE ELS PRODUCTES MOSTRATS EN LES TARGETES LI AGRADIN? Per exemple, 0% de probabilitat vol dir que no hi ha cap possibilitat que el producte li agradarà, mentre que el 100% de probabilitat vol dir que el producte li agradarà amb certesa.

Hi ha una probabilitat del _______% que el **producte A** m'**AGRADI**
Hi ha una probabilitat del _______% que el **producte B** m'**AGRADI**
Hi ha una probabilitat del _______% que el **producte C** m'**AGRADI**
Hi ha una probabilitat del _______% que el **producte D** m'**AGRADI**
Hi ha una probabilitat del _______% que el **producte E** m'**AGRADI**

31. Segons la seva opinió, amb quina PROBABILITAT CREU QUE ELS PRODUCTES MOSTRATS EN LES TARGETES SIGUIN SALUDABLES? Per exemple, 0% de probabilitat vol dir que no hi ha cap possibilitat que el producte sigui saludable, mentre que el 100% de probabilitat vol dir que el producte sigui saludable amb certesa.

Hi ha una probabilitat del _______% que el **producte A** sigui **SALUDABLE**
Hi ha una probabilitat del _______% que el **producte B** sigui **SALUDABLE**
Hi ha una probabilitat del _______% que el **producte C** sigui **SALUDABLE**
Hi ha una probabilitat del _______% que el **producte D** sigui **SALUDABLE**
Hi ha una probabilitat del _______% que el **producte E** sigui **SALUDABLE**

Nombre cabina____ Data___________

A continuació rebràs cinc mostres codificades i amb un ordre pre-establert. Si us plau, apunta't cada codi i tasta cada mostra individualment, agafant una mica de pa torrat i aigua entre mostra i mostra. Per a cada tast indica quant t'ha agradat el producte, posant una creu en el quadrat corresponent a la teva avaluació, des de "em desagrada extremadament" fins a "m'agrada extremadament".

	CODI MOSTRA	EM DESAGRADA EXTREMADAMENT	EM DESAGRADA MOLT	EM DESAGRADA	EM DESAGRADA UNA MICA	NI M'AGRADA NI EM DESAGRADA	M'AGRADA UNA MICA	M'AGRADA	M'AGRADA MOLT	M'AGRADA EXTREMADAMENT
**Mostra 1**										
**Mostra 2**										
**Mostra 3**										
**Mostra 4**										
**Mostra 5**										

N. cabina____ Data___________

Et presentem 5 etiquetes corresponents a cinc tipus diferents d'hamburgueses. Per a cada producte hauries d'indicar quant creus que et podria agradar, posant una creu corresponent a la teva avaluació amb una escala que va des de "em desagrada extremadament" fins a "m'agrada extremadament". Per a cada avaluació et demanem que et bases únicament en la informació indicada a continuació.

Producte A

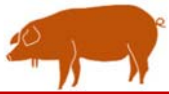

Hamburguesa Porc Negre Mallorquí

Carn de raça autòctona criada en sistema extensiu

Producte B

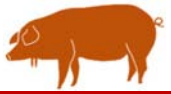

Hamburguesa Porc Negre Mallorquí

Carn de raça autòctona criada en sistema extensiu
Preparada amb una font natural de **fibra alimentària** que millora les nostres defenses

Producte C

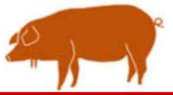

Hamburguesa Porc Negre Mallorquí

Carn de raça autòctona criada en sistema extensiu
Preparada amb una font natural d'**antioxidants** que ajuda a prevenir malalties cardiovasculars

Producte D

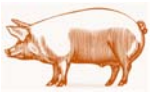

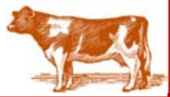

Hamburguesa mixta Porc i Vedella

Producte E

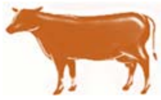

Hamburguesa Vedella

Carn selecta

	EM DESAGRADA EXTREMADAMENT	EM DESAGRADA MOLT	EM DESAGRADA	EM DESAGRADA UNA MICA	NI M'AGRADA NI EM DESAGRADA	M'AGRADA UNA MICA	M'AGRADA	M'AGRADA MOLT	M'AGRADA EXTREMADAMENT
**Producte A**									
**Producte B**									
**Producte C**									
**Producte D**									
**Producte E**									

N. cabina____ Data___________

A continuació, tornaràs a provar cada mostra de producte presentada anteriorment, amb la diferència que aquest cop et demanarem que avalues cada hamburguesa basant-te tant en les informacions indicades a continuació com en les característiques sensorials del producte. Recordeu-vos de provar cada mostra individualment i de prendre una mica de pa torrat i aigua entre mostra i mostra.

911

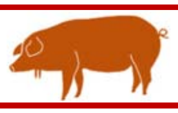

Hamburguesa Porc Negre Mallorquí

Carn de raça autòctona criada
en sistema extensiu

427

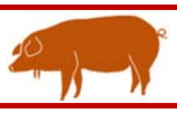

Hamburguesa Porc Negre Mallorquí

Carn de raça autòctona criada
en sistema extensiu
Preparada amb una font natural de
fibra alimentària que millora
les nostres defenses

333

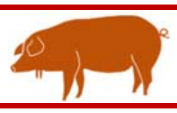

Hamburguesa Porc Negre Mallorquí

Carn de raça autòctona criada
en sistema extensiu
Preparada amb una font natural
d'**antioxidants** que ajuda a prevenir
malalties cardiovasculars

250

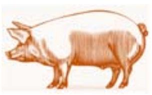

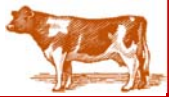

Hamburguesa mixta Porc i Vedella

541

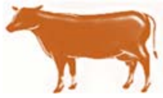

Hamburguesa Vedella

Carn selecta

	CODI MOSTRA	EM DESAGRADA EXTREMADAMENT	EM DESAGRADA MOLT	EM DESAGRADA	EM DESAGRADA UNA MICA	NI M'AGRADA NI EM DESAGRADA	M'AGRADA UNA MICA	M'AGRADA	M'AGRADA MOLT	M'AGRADA EXTREMADAMENT
**Mostra 1**										
**Mostra 2**										
**Mostra 3**										
**Mostra 4**										
**Mostra 5**										
